# Sedentary behavior and cancer–an umbrella review and meta-analysis

**DOI:** 10.1007/s10654-022-00873-6

**Published:** 2022-05-25

**Authors:** Rafael Hermelink, Michael F. Leitzmann, Georgios Markozannes, Kostas Tsilidis, Tobias Pukrop, Felix Berger, Hansjörg Baurecht, Carmen Jochem

**Affiliations:** 1 Department of Epidemiology and Preventive Medicine, University of Regensburg, Franz-Josef-Strauß-Allee 11, 93053 Regensburg, Germany; 2 Department of Internal Medicine II–Gastroenterology, University Hospital Munich, Munich, Germany; 3 Department of Hygiene and Epidemiology, University of Ioannina, Ioannina, Greece; 4 Department of Epidemiology and Biostatistics, Imperial College London School of Public Health, London, UK; 5 Department of Haematology and Internal Oncology, University Hospital of Regensburg, Regensburg, Germany

**Keywords:** Sedentary behavior, Meta-analysis, Umbrella review, Sitting time, Cancer prevention, Cancer mortality

## Abstract

Several systematic reviews and meta-analyses have summarized the association between sedentary behavior (SB) and cancer. However, the level of evidence and the potential for risk of bias remains unclear. This umbrella review summarized the current data on SB in relation to cancer incidence and mortality, with a particular emphasis on assessing the risk of bias. We searched PubMed, Web of Science and Cochrane Database for systematic reviews and meta-analyses on the association between SB and cancer incidence and mortality. We also searched for recent observational studies not yet included in existing meta-analyses. We re-calculated summary risk estimates for cancer incidence and mortality using random effects models. We included 14 meta-analyses covering 17 different cancer sites from 77 original studies. We found that high SB levels increase the risk for developing ovarian, endometrial, colon, breast, prostate, and rectal cancers, with relative risks of 1.29 (95% confidence interval (CI) = 1.08–1.56), 1.29 (95% CI = 1.16–1.45), 1.25 (95% CI = 1.16–1.33), 1.08 (95% CI = 1.04–1.11), 1.08 (95% CI = 1.00–1.17), and 1.07 (95% CI = 1.01–1.12), respectively. Also, we found an increased risk of cancer mortality of 1.18 (95% CI = 1.09–1.26). Most associations between SB and specific cancer sites were supported by a “suggestive” level of evidence. High levels of SB are associated with increased risk of several types of cancer and increased cancer mortality risk.

**Supplementary Information:**

The online version contains supplementary material available at 10.1007/s10654-022-00873-6.

## Introduction

Worldwide, the prevalence of cancer has risen sharply in recent years, and cancer contributes to a large burden of disease [1, 2]. To decrease cancer incidence, prevalence and mortality, a preventive approach is crucial. Whereas known risk factors such as tobacco use and alcohol consumption should be avoided, protective factors such as physical activity, a healthy diet and a healthy body weight should be strengthened [3]. Sedentary behavior has been shown to increase cancer incidence and mortality [4]. Sedentary behavior is defined as “any waking behavior characterized by an energy expenditure ≤ 1.5 metabolic equivalents, while in a sitting, reclining or lying posture” [5] and is a highly prevalent behavior in our daily lives [6]. Adults spent approximately 8.2 h per day with sitting behaviors [6].

Findings of existing observational studies on the association between sedentary behavior and cancer have been summarized by a growing number of systematic reviews and meta-analyses. Specifically, sedentary behavior has been shown to increase the risks of colorectal, breast, ovarian and endometrial cancers [7–11]. However, the potential risk of bias in previous studies has not yet been well investigated and therefore, the level of evidence remains unclear. In addition, some meta-analyses reported high levels of statistical heterogeneity for some of the associations, particularly the relation with colorectal cancer incidence. Such statistical heterogeneity may weaken the level of evidence.

The primary aim of this umbrella review was to critically analyze existing systematic reviews and meta-analyses of sedentary behavior in relation to risk of various cancers and cancer mortality. We performed an updated meta-analysis of every association between sedentary behavior and cancer incidence previously examined and we rated the levels of evidence regarding the individual associations. We additionally performed a second screening for individual studies that were not already incorporated in the included systematic reviews or meta-analyses to provide the most accurate and up-to-date evidence from the literature.

## Methods

### Literature search

We conducted a comprehensive literature search from inception to October 2021 by screening PubMed, Web of Science, and the Cochrane Database of Systematic Reviews for systematic reviews and meta-analyses that investigated the association between sedentary behavior and risk of cancer incidence and mortality. Supplementary Table S1 shows the complete search strategy for PubMed. Similar search algorithms were used for the other databases. In addition, we hand-searched the reference lists of the eligible studies.

To identify individual studies that had not yet been included in previous systematic reviews and meta-analyses, we performed a comprehensive literature search of relevant observational studies (including cohort and case–control studies) published between January 2014 and October 2021 (Supplementary Table S2). A search for missing individual studies prior to January 2014 was not performed since that time period was already covered by underlying meta-analyses. The literature search and all methodological steps of the umbrella review were defined a priori in an internal protocol (that is not publicly available).

### Selection of reviews

We defined inclusion and exclusion criteria a priori. We only considered systematic reviews and meta-analyses published in English that investigated the association between sedentary behavior and risk of cancer incidence or all-cancer mortality. Furthermore, we included one systematic review [12] that additionally investigated post-diagnosis sedentary behavior in relation to colorectal cancer-specific mortality. We included systematic reviews that performed a quantitative analysis and provided a summary effect measure as well as the corresponding data from individual studies. We excluded narrative reviews or systematic reviews that did not contain a quantitative synthesis. We further excluded meta-analyses if individual studies were already included or if physical inactivity was used as reference.

**Fig. 1:**
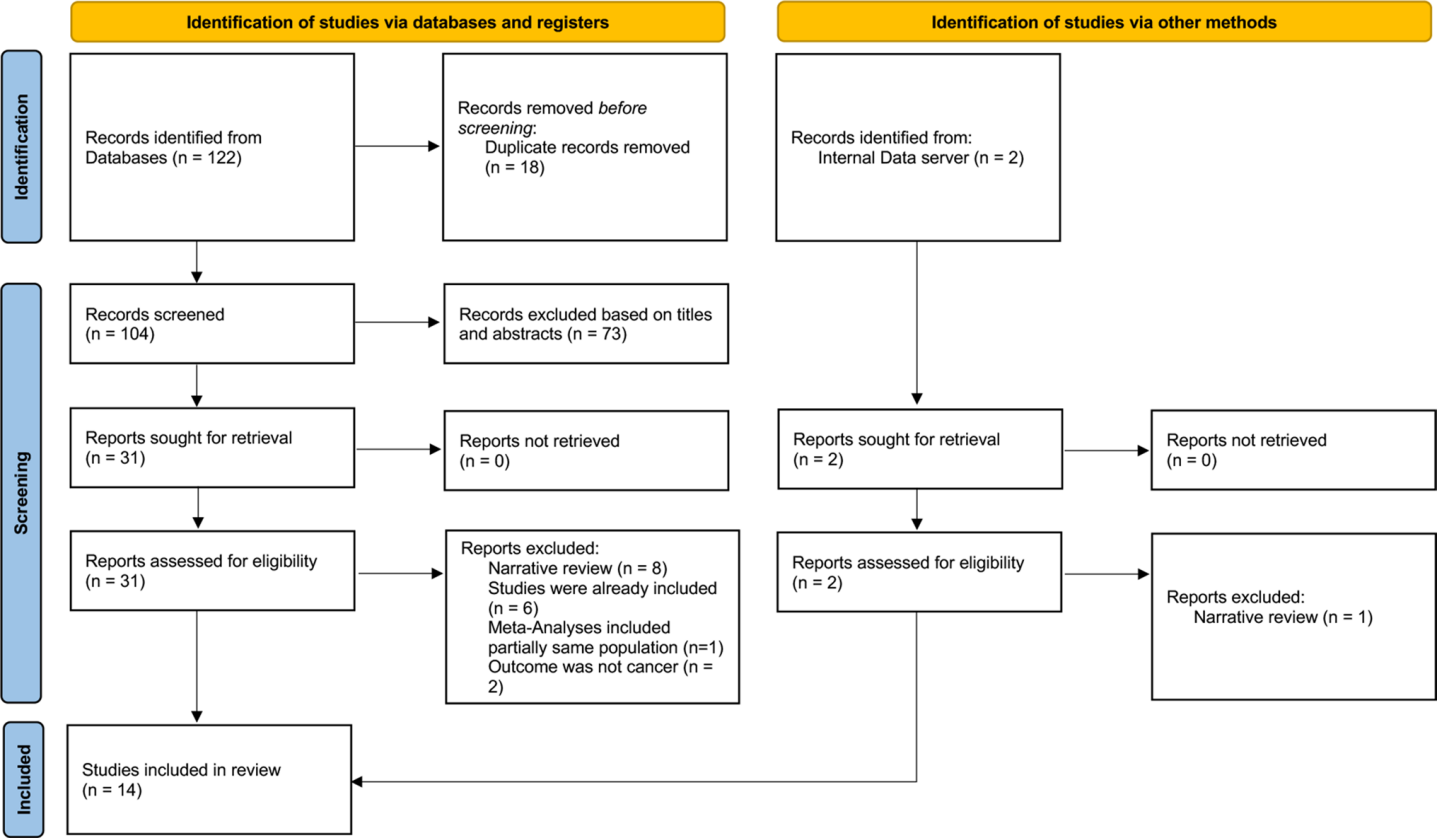
Flow diagram of literature search and study selection for systematic reviews and meta-analyses

Two researchers (RH and CJ) independently screened titles, abstracts, and full texts; removed duplicates; and selected the eligible reviews. A third researcher (ML) settled disagreements between the two researchers (Fig. 1). If several meta-analyses examined the same cancer site and included the same observational studies, we included the most recent meta-analysis. If several meta-analyses examined the same cancer site but included different individual studies (i.e., for colon, rectum and breast cancers), we included more than one meta-analysis for a specific cancer site in order to ensure that all existing individual studies were included in the umbrella review. To avoid overlap of studies, data analyses were performed separated by review. Supplementary Table S3 shows the included and excluded reviews as well as the reasons for exclusion.

### Selection of studies

**Fig. 2:**
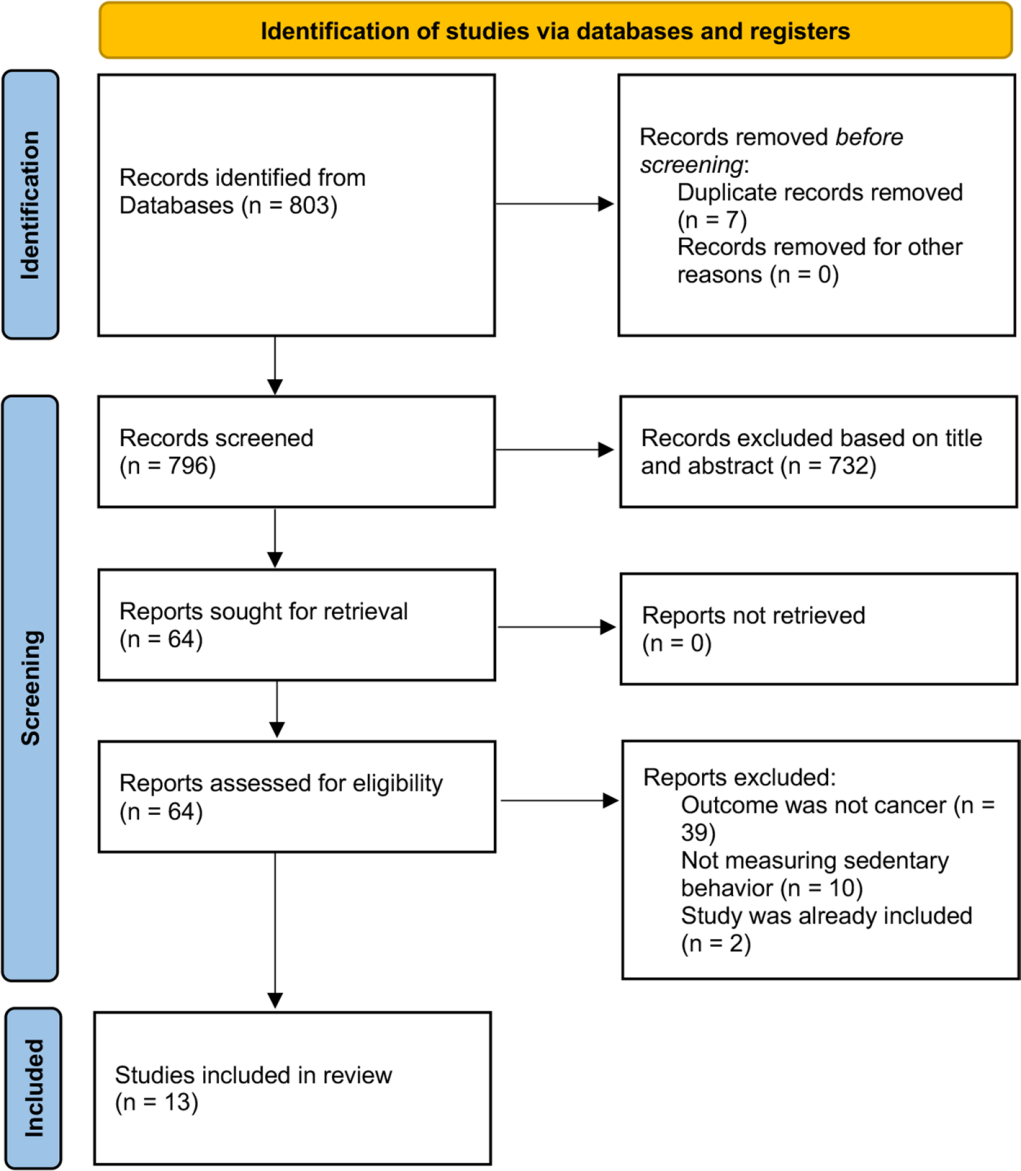
Flow diagram of literature search and study selection for additional individual studies

Since there was no up-to-date meta-analysis available for every single association at the time this umbrella review was conducted, we undertook a second screening without restriction to systematic reviews or meta-analysis to include potentially missed observational studies (Fig. 2). We used the same screening criteria and examined relevant databases from January 2014 to October 2021 (Supplementary Table 2). New individual studies included in our meta-analyses needed to be observational in nature, investigate the association between any type of sedentary behavior and cancer incidence or mortality, report summary risk estimates and corresponding 95% CI, and perform adequate statistical adjustment. We excluded individual studies that used the terms “sedentary” or “sitting” to define the reference group of physical activity categories, or studies that represented a mere update of a previous study.

### Assessment of methodological quality

Two researchers (RH and CJ) independently assessed the methodologic quality of the included meta-analyses using “A Measurement Tool to Assess Systematic Reviews–2” (AMSTAR-2, [13]) (Supplementary Table S4), a validated tool suitable for systematic reviews of non-randomized intervention studies. Subsequently, ratings were compared and disagreements were resolved by consensus after further discussion with a third party (ML).

### Data extraction

We extracted the following data from the included meta-analyses: first author’s name, year of publication, number of studies included, sample size, type of sedentary behavior (total sitting, occupational sitting, recreational sitting, TV-viewing time), type of cancer, and summary risk estimates (relative risk (RR), hazard ratio (HR) or odds ratio (OR)) with corresponding 95% confidence intervals (CI). For each individual study included in the meta-analyses, we extracted the first author’s name, year of publication, study design (case–control or cohort study), sex, numbers of cases and controls (for case–control studies), numbers of cases and sample size (for cohort studies), type of sedentary behavior, summary risk estimate (RR, HR, or OR), and 95% CI. Furthermore, we extracted the study geographic region and statistical adjustments considered. If available, we also extracted the results of subgroup and dose–response analyses. When more than one meta-analysis was included for a specific cancer site, we only extracted the data from the individual studies to avoid including partly the same population. Two authors (RH and CJ) independently performed data extraction and resolved inconsistencies by consensus.

### Statistical analysis

#### Main analysis

The primary aim of this umbrella review was to examine the association between ‘any sedentary behavior’ (i.e., total sitting, occupational sitting, recreational sitting, and TV-viewing time) and cancer incidence. If individual studies investigated more than one type of sedentary behavior, we selected the summary estimates in the following order: total sitting, recreational sitting, occupational sitting and TV-viewing time. We re-calculated summary risk estimates (represented as RR, HR, or OR) for risk of cancer at individual sites as well as for all-cancer mortality and for cancer-specific mortality using random effects models. Because the absolute risks of the studied outcomes are expected to be low in the general population, the 3 measures of association (OR, RR, HR) are also expected to produce comparable estimates. Therefore, all risk estimates were interpreted as relative risks (RRi) for simplicity [14]. For data analysis, we combined the individual studies from the included meta-analyses with newly screened individual studies. To account for the fact that case–control studies show a lesser degree of validity than cohort studies, we conducted separate meta-analyses of cohort studies only. All statistical analyses were performed using R, version 4.0.2. [15].

#### Stratified, subgroup, and influence analyses

For each cancer entity, we performed meta-regression random effects meta-analysis to assess potential heterogeneity by sex (for non-sex-specific cancers), study geographic region, study design, sedentary behavior domain, and number of adjustment factors. Furthermore, we investigated the influence of adjusting for specific confounders, namely, body mass index (BMI), smoking, alcohol consumption, and family history of cancer. If reasonable, we conducted analyses stratified by menopausal and hormone use status. We performed influence diagnostics to detect potential outliers that strongly influence heterogeneity or effect size [16, 17]. Furthermore, we implemented the Graphic Display of Heterogeneity (GOSH) plot, a method proposed by Olkin et al. [18]. If outliers were discovered, we conducted sensitivity analyses excluding the outliers to see whether the observed heterogeneity could be significantly reduced.

#### Between-study heterogeneity

To detect possible heterogeneity between studies, we applied the *I*^*2*^ statistic, which reflects the proportion of the total variation across studies beyond chance [19]. An *I*^*2*^ value > 50% was considered an indication of high heterogeneity. We also calculated the 95% prediction intervals [20]. The prediction intervals further take into account the between-study heterogeneity and estimate the range of effects that would be expected in a future study investigating the same association.

#### Publication bias, small study effect and excess significant biases

To detect potential publication bias, we created funnel plots for our calculated meta-analyses [17]. To assess whether asymmetry in the funnel plot was due to small study effects, we performed the regression asymmetry test proposed by Egger and colleagues [21]. Small study effect bias was considered present when Egger’s test *p* value was < 0.10 and the effect size of the largest study (smaller standard error (SE)) of a meta-analysis was lower than the meta-analysis random effects estimate [22]. When Egger’s test showed significant asymmetry, we performed the trim and fill test to estimate the actual effect size if the potential “missing” studies had been published [23]. Furthermore, we performed the excess significance test to evaluate whether the number of statistically significant studies with *p* < 0.05 included in each individual meta-analysis differed from the expected number of significant studies. An excess of statistically significant findings may imply selective reporting bias in a meta-analysis. The expected number of significant associations was calculated based on the sum of the statistical power estimates for each component study, based on a noncentral *t* distribution against a plausible effect size, which was defined as the effect size of the largest and most precise study (smallest standard error) in each meta-analysis. If the number of statistically significant studies in the test was disproportionately high (*p* < 0.1), an excess of significant studies was assumed [24, 25].

### Grading the evidence

It has become common practice to classify meta-analyses by their level of evidence and to perform umbrella reviews in order to provide the highest quality of evidence from existing meta-analyses [26]. In line with previous umbrella reviews[27–30], we classified the evidence of the included meta-analyses with statistically significant (*p* < 0.05) results as convincing, highly suggestive, suggestive, or weak, following the criteria by Fusar-Poli et al. [14]. Table 1 shows the criteria for each category of evidence. Briefly, a strong association was reported when the random-effects *p* < 10^−6^ (a threshold considered to substantially reduce false positive findings) [31–33], the meta-analysis included > 1000 cases, there was no substantial between-study heterogeneity, the prediction intervals excluded the null value, and there was no indication of small study or excess significance bias. A highly suggestive association was supported by a *p* < 10^−6^, > 1000 cases, and the largest study in the meta-analysis presenting a nominally significant effect. Associations with suggestive evidence had > 1000 cases and a *p* < 10^−3^. All other nominally statistically significant associations (i.e., *p* < 0.05) were denoted as weak evidence. This grading scheme allows for an objective, standardized classification of the level of evidence [14, 34] and has been shown to provide consistent results with other grading schemes, especially in the field of cancer epidemiology [29].

**Table 1:** Criteria used for grading the level of evidence of studies examining sedentary behavior and cancer incidence or cancer mortality

Evidence		Elevated Risk	
	Criteria	Cohort and Case–Control	Cohort
Convincing (Class I)	*p* < 10^–6^, > 1000 cases, I^2^ < 50%, 95% prediction interval excluding the null, no small-study effect* and no excess significance bias†	None	None
Highly suggestive (Class II)	*p* < 10^–6^, largest study with a statistically significant effect and class I criteria not met	All-cancer mortality	All-cancer mortality
Suggestive (Class III)	> 1000 cases, *p* < 10^−3^ and class I–II criteria not met	Colon cancer, Endometrial cancer, Breast cancer, Rectal cancer, Ovarian Cancer	Colon cancer, Endometrial cancer, Breast cancer
Weak (Class IV)	*p* < 0.05 and class I–III criteria not met	Prostate cancer	Rectal cancer, Prostate cancer, Ovarian Cancer
Non-significant (Class V)	*p* ≥ 0.05	Lung cancer, Gastric cancer, Esophageal cancer, Testicular cancer, Renal cell cancer, Non-Hodgkin Lymphoma, Gallbladder cancer, Head and Neck cancer, Liver cancer, Melanoma, Multiple Myeloma, Pancreatic cancer	Lung cancer, Gastric cancer, Esophageal cancer, Testicular cancer, Renal cell cancer, Non-Hodgkin Lymphoma, Gallbladder cancer, Head and Neck cancer, Liver cancer, Melanoma, Multiple Myeloma, Pancreatic cancer

*Small study effect is based on the p-value from Egger’s regression asymmetry test (*p* ≤ 0.1)

^†^Based on the *p*-value (*p* < 0.1) of the excess significance test using the largest study (smallest standard error) in a meta-analysis as the plausible effect size

## Results

### Description and characteristics of the included meta-analyses

Our search of electronic databases and hand-searching of reference lists yielded a total of 122 studies. After the exclusion of 91 reviews that did not meet our inclusion criteria, a total of 14 systematic reviews and meta-analyses were eligible for our umbrella review (Fig. 1). Ten of the 14 included systematic reviews examined the association of sedentary behavior and cancer incidence, whereas five systematic reviews examined all-cancer mortality, with one meta-analysis [35] investigating both cancer incidence and mortality. Supplementary Table S3 shows the characteristics of the included systematic reviews and meta-analyses, and Supplementary Table S4 shows the detailed scoring of each systematic review based on the AMSTAR-2 rating. None of the meta-analyses explained their selection of the study designs for inclusion in the review (Item 3, Supplementary Table S4) and none reported the source of funding (Item 10, Supplementary Table S4). Furthermore, the majority of reviews did not provide a list of excluded studies or justify the exclusions (Item 7, Supplementary Table S4).

Our literature search for individual observational studies that had not yet been included in published meta-analyses revealed thirteen additional and non-overlapping studies [36–48]; two studies on lung cancer incidence, three on colorectal cancer incidence, one on ovarian cancer incidence, one on endometrium cancer incidence, one on breast cancer incidence, one on esophageal cancer incidence, one on melanoma incidence, two on all-cancer mortality and one on several types of cancer (Fig. 2).

For cancer incidence of individual sites, we included ten [7–9, 11, 35, 49–53] meta-analyses with a total of 212,707 cancer cases and 17 different cancer sites from 77 individual studies (50 cohort and 27 case–control studies). Of those eleven meta-analyses, ten showed a statistically significant association with cancer incidence.

The association between sedentary behavior and all-cancer mortality was examined by five [12, 35, 54–56] systematic reviews and meta-analyses and one new individual study, yielding a total of 50,406 reported cancer deaths from 17 cohort studies. In addition, three [57–59] cohort studies examined the association between post-diagnosis sedentary behavior and colorectal cancer-specific mortality, and one study [60] investigated the association between overall sedentary behavior and colorectal cancer-specific mortality. The association for prostate cancer-specific mortality was examined by three [61–63] cohort studies included in the meta-analysis by Berger et al [49].

All 14 meta-analyses included in our umbrella review (Supplementary Table S3) calculated summary effect estimates and corresponding 95% CIs using a random effects model. Whereas all meta-analyses reported the number of cases, none performed an excessive significance test and none calculated prediction intervals. By comparison, *I*^*2*^ heterogeneity estimates (n = 14; 100%), funnel plots (n = 13; 93%), and small study effects (n = 12; 86%) were reported by the majority of included meta-analyses.

## Summary effect size and robustness of evidence

**Fig. 3:**
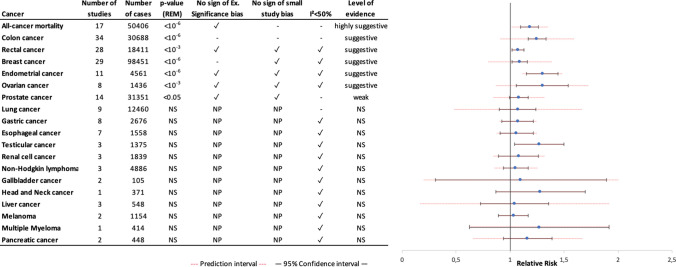
Grading the level of evidence of both case–control and cohort studies for the relationship between sedentary behavior and cancer incidence or cancer mortality. Number of studies is referred to the number of studies included in the individual meta-analysis. Number of cases is referred to the number of cancer conditions. Small study bias is considered positive if the p-value in Egger's test is less than 0.10. The excess significance bias is considered positive if the number of significant studies is greater than the number of significant studies expected (based on the largest study with the smallest SE) and the p-value is less than 0.10. Abbreviations: REM random effect model, NS not statistically significant, NP not performed

**Fig. 4:**
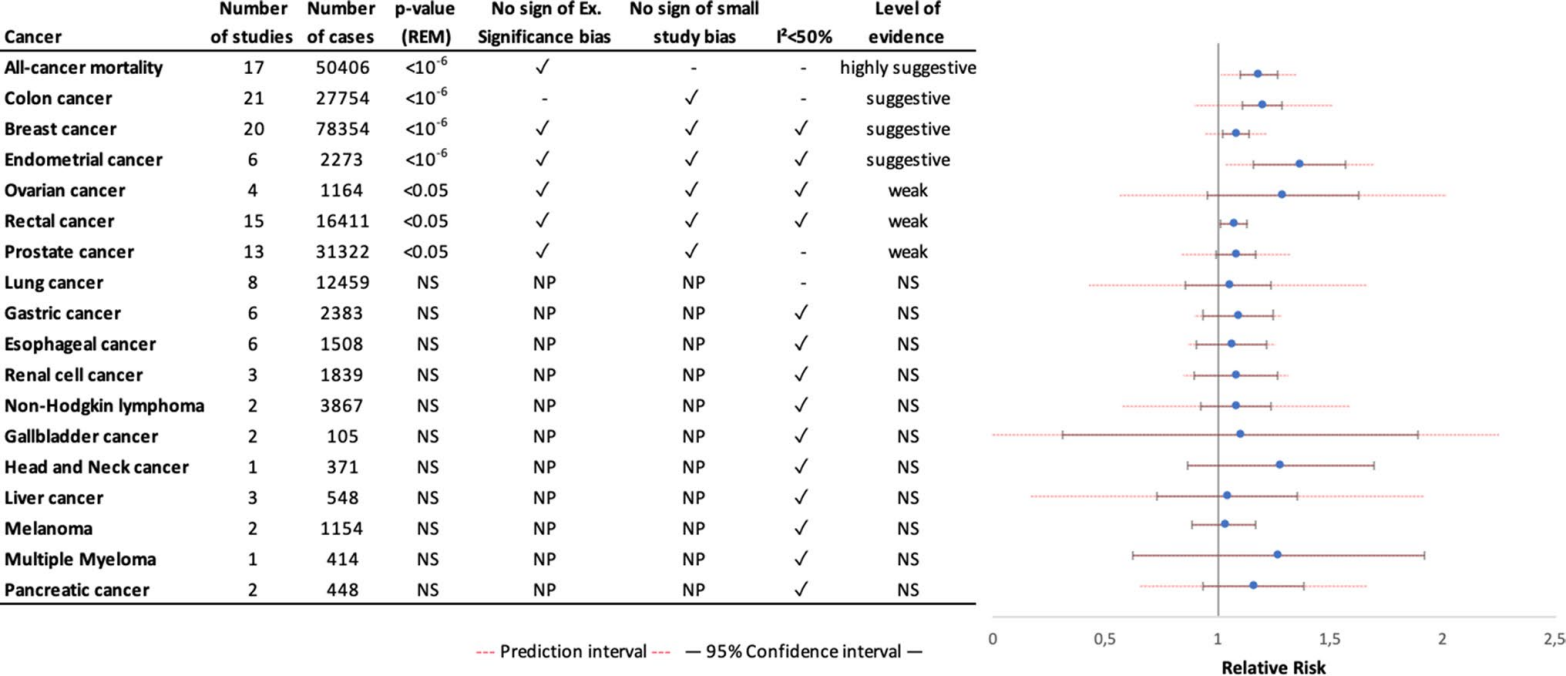
Grading the level of evidence of cohort studies for the relationship between sedentary behavior and cancer incidence or cancer mortality. Number of studies is referred to the number of studies included in the individual meta-analysis. Number of cases is referred to the number of cancer conditions. Small study bias is considered positive if the p-value in Egger's test is less than 0.10. The excess significance bias is considered positive if the number of significant studies is greater than the number of significant studies expected (based on the largest study with the smallest SE) and the p-value is less than 0.10. Abbreviations: REM random effect model, NS not statistically significant, NP not performed

After combining all individual studies (extracted from the included meta-analyses and combined with newly identified individual studies) in our newly performed meta-analyses, the association between sedentary behavior and cancer incidence was statistically significant in six of 17 (35%) cancer sites (ovarian, breast, colon, endometrial, rectal and prostate cancers) (Figs. 3 and Fig. 4). Following the criteria reported in Table 1, the associations between sedentary behavior and breast cancer (RR = 1.08; 95% CI = 1.04–1.12), colon cancer (RR = 1.25; 95% CI = 1.16–1.33), rectal cancer (RR 1.07; 95% CI 1.01–1.12), endometrial cancer (RR = 1.29; 95% CI = 1.15–1.44) and ovarian cancer (RR = 1.29; 95% CI = 1.17–1.54) were supported by a suggestive level of evidence, whereas the association with prostate cancer (RR = 1.08; 95% CI 1.00–1.17) showed a weak level of evidence. When including only cohort studies in our meta-analysis, the levels of evidence were lowered for rectal cancer incidence and ovarian cancer incidence (Supplementary Tables S5 and S6). Furthermore, there was highly suggestive evidence that sedentary behavior is associated with increased risk of all-cancer mortality (RR = 1.18; 95% CI = 1.09–1.26).

Three individual studies additionally examined the relationship between post-diagnosis sedentary behavior and colorectal cancer-specific mortality. Sedentary behavior performed after colorectal cancer diagnosis led to increased colorectal cancer-specific mortality (RR = 1.61, 95% CI = 1.23–2.11). Furthermore, one study conducted among Japanese Population [60] found that sedentary behavior led to an overall increased risk of colorectal cancer-specific mortality (RR = 1.33, 95% CI = 1.02–1.73). Finally, there was a non-significant increase in overall prostate cancer-specific mortality (RR = 1.14, 95% CI = 0.94–1.38) after pooled-analysis from three individual studies was performed in the meta-analysis by Berger et al.

One meta-analysis [11] reported that the association between sedentary behavior and breast cancer and was positive in postmenopausal women (RR = 1.20; 95% CI = 1.00–1.44), whereas it was null in premenopausal women (RR = 1.04; 95% CI = 0.84–1.32).

Tables S5 and S6 show the robustness of evidence grading for the meta-analyses of the associations between sedentary behavior and risk of individual cancers. In addition to the results described above, we could not find a statistical significant relation of sedentary behavior to increased risks of lung cancer, gastric cancer, esophageal cancer, renal cell cancer, testicular cancer, liver cancer, non-Hodgkin lymphoma, pancreatic cancer, melanoma, multiple myeloma and head and neck cancer.

### Publication bias, small study effect and excess significant biases

Visual inspection of the funnel plots of the seven statistically significant meta-analyses revealed asymmetry of the association between sedentary behavior and colon cancer incidence and all-cancer mortality, and Egger´s test indicated the existence of small study effects for the two meta-analyses (*p* < 0.10). After applying the trim and fill test, the positive associations in the two meta-analyses were weakened, supporting the presence of small study effects (Supplementary Table S5).

When only cohort studies were included in the meta-analyses, the association between sedentary behavior and colon cancer no longer showed small study effects (Egger´s *p* ≥ 0.1) (Supplementary Table S6). Furthermore, if only case–control studies published after 1999 were considered in the meta-analysis, the small study effects bias was no longer present, without altering the summary risk estimate (RR = 1.24; 95%CI = 1.16–1.32, Egger’s *p* > 0.1). This time cut point was chosen because it is only in the past 20 years that a comparable definition of sedentary behavior has emerged.

Two meta-analyses including both cohort and case–control studies on the associations with colon and breast cancer incidence showed an excess of significant results beyond the expected number of significant studies, suggesting publication bias. When we restricted the meta-analyses to cohort studies, the excess significance bias for the association with breast cancer disappeared, while the excess significance for the association with colon cancer remained evident (Supplementary Tables S5 and S6).

### Between-study heterogeneity and prediction intervals

Ten of the 17 (59%) associations with cancer incidence showed lower levels of statistical heterogeneity, whereas the remaining seven associations showed a greater level of statistical heterogeneity (Supplementary Tables S5 and 6). When considering only the seven statistically significant results (six for cancer incidence and one for all-cancer mortality), the associations with colon and prostate cancer incidence as well as with all-cancer mortality showed signs of higher statistical heterogeneity between studies (*I*^*2*^ > 50%). When calculating the prediction intervals, the null could not be excluded in three of the seven significant associations (breast, colon and prostate cancer; Supplementary Tables S5 and S6).

### Stratified, subgroup, and influence analyses

We performed stratified analyses of endpoints with statistically significant associations (Supplementary Tables S7-S13). The association between sedentary behavior and colon cancer incidence showed a statistically significant difference in the magnitude of risk according to study design, where the risk increase was greater when only case–control studies were included. For the remaining associations, differences between study design subgroups were less consistent. Furthermore, there were relevant differences when analyzing the various geographic regions in which the studies were conducted. In particular, studies from the Asian region showed divergent results in the majority of meta-analyses. Particular attention was paid to whether studies were adjusted for BMI. There was no consistent result. In some instances, the association for certain cancers (e.g., endometrial cancer) was null in the analysis that was adjusted for BMI, although differences between studies were not statistically significant.

Additionally, we performed influence diagnostics and GOSH plots to detect and remove outliers and we re-ran our models to assess whether observed heterogeneity could be reduced. For ovarian cancer incidence, we detected one outlier [64]. After removing that study from the meta-analysis, heterogeneity was reduced without changing the effect size.

## Discussion

### Primary findings

The aim of this umbrella review was to investigate the evidence regarding the associations between sedentary behavior and cancer incidence and all-cancer mortality. To this end, we combined data for 17 individual cancer sites from more than 70 distinct studies, including a total of over 200,000 cancer cases. We found statistically significant positive associations between sedentary behavior and risk of cancer incidence for six cancer sites (ovarian, endometrial, colon, breast, rectal and prostate cancers) as well as a statistically significant positive association with all-cancer mortality. Apart from the identified meta-analyses, the evidence from literature of a number of narrative reviews without quantitative synthesis is largely in line with the evidence reported in our review.

The association between sedentary behavior and ovarian cancer showed a suggestive level of evidence. However, that level of evidence was lowered to a weak level of evidence when including only cohort studies. As some of the included studies contributed less than ten cancer cases to the summary of evidence, the power of the evidence is limited. Nevertheless, the observed evidence regarding sedentary behavior and ovarian cancer is an important finding, in particular because the guidelines put forth by the International Agency for Research on Cancer (IARC [65]), the World Cancer Research Fund International (WCRF [66]), and the Physical Activity Guidelines Advisory Committee Scientific Report (PAGA [67]) have not yet identified a meaningful association between sedentary behavior and ovarian cancer risk. Furthermore, our study indicated a suggestive level of evidence regarding positive associations between sedentary behavior and cancers of the endometrium, colon, breast, and rectum.

Finally, among our statistically significant results, only the association with prostate cancer incidence was supported by a weak level of evidence, mainly because the p-value was just below the 0.05 threshold, while there was no sign of small study effects or publication bias. Even if this is a completely new finding, the association remains weak and could be explained by a lack of sufficient adjustments and residual confounding. Complementary, the trim and fill test revealed six missing studies. Further studies are required to potentially establish a higher level of evidence regarding the sedentary behavior and prostate cancer relation.

In our umbrella review, we paid particular attention to assessing the risk of bias and we applied numerous statistical methods to evaluate the level of evidence regarding individual associations. To assess the credibility of evidence, we used a series of statistical tests to obtain hints of bias in the literature. Other criteria for grading the evidence (e.g., Bradford Hill or GRADE) have been proposed by various authors [68, 69]. The criteria we used were aimed at examining the potential for biases. However, caution is needed when interpreting the criteria for grading the evidence used in this umbrella review. It is important to keep in mind that it is not possible to estimate the exact extent or source of bias that affects the evidence and that there is no study that has evaluated the validity of the criteria used in this umbrella review theoretically such as using a simulation study. Nevertheless, empirical evidence shows that this grading scheme works remarkably well compared to other grading schemes and has been used in numerous umbrella reviews [27–30].

The association of sedentary behavior to colon cancer incidence and all-cancer mortality showed signs of heterogeneity and small study effect bias. When considering cohort studies only, small study bias remained for the association with all-cancer mortality but was no longer present for the association with colon cancer incidence, indicating a possible bias in case–control studies of colon cancer. Also, for colon cancer we found a statistically significant difference in the summary risk estimate according to study design, with a more pronounced risk increase in case–control than cohort studies (47% versus 20%). This may be explained by a disproportionately high number of unadjusted and dated case–control studies, which used heterogeneous assessments of sedentary behavior and included small numbers of cases. In fact, the small study effect disappeared when case–control studies that were published before 1999 were excluded (n = 9). Excluding dated case–control studies only slightly weakened the summary effect estimate (RR = 1.24) but additionally reduced the heterogeneity from 51 to 32%, which indicates improved data quality for case–control studies published from the year 2000 onward. However, the power to detect small study effects was limited due to a restricted (n < 10) number of included studies on the association with ovarian cancer incidence and endometrial cancer incidence when only cohort studies were considered.

In addition to uncover potential small study effects, we focused on examining potential excess significance bias. Of the seven significant meta-analyses, there was excess significance bias regarding the associations of sedentary behavior with colon and breast cancers. If only cohort studies were considered, that bias persisted for the association with colon cancer incidence, confirming publication bias regarding the literature on sedentary behavior and colon cancer incidence. Interestingly, the excessive significance bias disappeared when only cohort studies were considered for breast cancer incidence, suggesting an excess number of significant case–control studies. However, the low amount of studies included in the analyses of endometrial and ovarian cancer incidence limited the power to detect excessive significance bias for these associations.

The associations between sedentary behavior and cancers of the lung, stomach, esophagus, testes, kidney, gallbladder, head and neck, liver, skin, pancreas, and non-Hodgkin lymphoma and multiple myeloma were statistically non-significant. For lung, esophageal and gastric cancer, there were a considerable number of available studies (nine, eight and seven individual studies, respectively) compared to the remaining cancer sites, for which data remains limited. For example, only one study investigated the association with multiple myeloma.

The WCRF report in its section on sedentary behavior mentions only the association between sedentary behavior and endometrial cancer, classifying it as "limited evidence." The IARC [65] and PAGA reports [67] classify the association between sedentary behavior and risk of colorectal and endometrial cancer as "limited evidence", with the IARC report further adding breast cancer incidence to that group. None of the three reports mention the association of sedentary behavior to ovarian or prostate cancer, the levels of evidence of which we were able to classify in our umbrella review.

Although IARC guidelines state that there is limited evidence for a borderline statistically significant positive association between sedentary behavior and lung cancer, a relation known for its potential for confounding by smoking, we found a null association in our analyses. The difference in findings may be attributed to the fact that the meta-analyses conducted to date had differences in inclusion criteria or prioritization of exposure type. Furthermore, the meta-analysis on which the IARC guidelines are based [7] was published in 2014, with only four observational studies included. With our updated analysis, we were able to detect four additional observational studies, resulting in a non-significant 7% risk increase for lung cancer incidence (RR = 1.07; 95% CI = 0.91–1.25) for high versus low sedentary behavior.

An additional focus of our umbrella review was the association between sedentary behavior and all-cancer mortality. We found that sedentary behavior was associated with a statistically significant 18% increased risk of all-cancer mortality. When applying the criteria listed in Table 1, the level of evidence appeared highly suggestive. There was clear evidence of heterogeneity across studies (*I*^*2*^ = 57%) and signs of small study effects (Egger´s *p* < 0.01). Furthermore, the meta-analysis by Patterson et al. [54] performed a dose–response analysis, where the summary linear estimate of the association between sedentary behavior (hours/day) was 1.03 (1.02–1.04) in individuals exposed to TV-viewing time, while the association with total sedentary behavior was not statistically significant. When adjusted for physical activity, the summary linear estimate was lowered to 1.02 (1.01–1.03) [54], confirming results showing that physical activity attenuates the sedentary behavior and all-cancer mortality association [55]. The results persisted across numerous subgroup analysis (Supplementary table S8).

Another aspect that has received more attention in recent years is the relationship between sedentary behavior and colorectal cancer-specific mortality. In our study, post-diagnosis sedentary behavior led to a risk increase of 61% for colorectal cancer-specific mortality, suggesting a contribution of sedentary behavior to worse survival after cancer diagnosis. However, caution is needed when analyzing the data for sedentary behavior and all-cancer mortality, as well as for cancer-specific mortality. Most included studies compared high vs low analyses and thus, the effect estimate may be inflated compared to a linear analysis. Based on the meta-analysis by Patterson et al. [54], an increased risk of 1.03 per hour of sedentary behavior/day is assumed. In our study, the estimate was 1.18, which would correspond to 5.5 h/day of sedentary behavior. Although most of the included studies used similar or higher cutoffs, few are based on lower amount of sedentary behavior/day.

To further explore potential sources of heterogeneity, we performed stratified analyses, influence analyses and outlier analyses. The most significant differences were found in sub-analyses stratified by geographic region and study design (Supplementary Table S7-S13). Since a large proportion of case–control studies included in our meta-analyses were from the Asian region, this could also explain the differences in results between study geographic regions. For most associations, an increased risk of developing cancer was shown in the TV-viewing subgroup. Even if differences were not statistically significant, relying on TV-viewing time as measure of sedentary behavior could lead to an overestimation of effects. Finally, within BMI subgroups, the association between sedentary behavior and cancer was null for some cancer entities. Although the difference between subgroups was not significant, this finding underlines the importance of adjusting for obesity.

An additional aim of the current study was to screen for individual studies not yet included in a previous meta-analysis. A total of thirteen new studies examining the association between sedentary behavior and cancer incidence or mortality were identified and were included in our analysis. There were no more than two additional studies for a given cancer site. No relevant changes in the summary estimates and no risk of bias were found when comparing the summary estimates with and without the newly added individual studies.

### Potential biologic mechanisms

In recent years, there has been an increased focus on the molecular and physiological mechanisms through which sedentary behavior may lead to cancer. It has been shown that sedentary behavior leads to obesity [70, 71], a condition that increases the risks of various types of cancer, including endometrial, esophageal, renal and pancreatic adenocarcinomas; hepatocellular carcinoma; gastric cardia cancer; meningioma; multiple myeloma; colorectal, postmenopausal breast, ovarian, gallbladder and thyroid cancers [29, 72, 73]. In postmenopausal women, adipocytes (localized mainly in fat tissue) are the main source of aromatase, an enzyme which stimulates the production of estrogens from androgens [74]. This mechanism causes an increased estrogen-induced proliferation of the endometrial mucosa, leading to an increased risk of gynecological neoplasia. Additionally, obesity reduces the production of sex hormone binding globulin (SHBG), which leads to increased circulating levels of free estrogen [75]. Sedentary behavior also leads to increased estrogen levels independent of obesity, which could contribute to the development of gynecological neoplasia [71].

Another possible mechanism that leads to increased cancer risk is the association of sedentary behavior and peripheral insulin resistance [76], the most important causal risk factor for type 2 diabetes [77]. Type 2 diabetes leads to increased levels of insulin-like growth factor-1 (IGF-1) and blood glucose. These have a well-known mitogenic effect, increasing the risk of developing various types of cancer, including cancers of the breast, endometrium, colon, liver, esophagus, kidney, and pancreas [78–81]. Finally, both sedentary behavior and obesity lead to increased systemic chronic inflammation [82, 83]. Systemic chronic inflammation is a well-known independent risk factor for the development of cancer [84].

### Strengths and limitations

To our knowledge, this is the first umbrella review examining the association between sedentary behavior and cancer incidence and all-cancer mortality, thus making a novel contribution to the literature. Since some of the included meta-analyses are no longer up-to-date and lack more recent studies, we conducted an additional screen to find new individual studies. This allowed us to present an up-to-date analysis of all cancer sites. We also evaluated all included meta-analyses according to objective criteria (AMSTAR-2). Furthermore, by performing the excessive significance bias test and prediction interval calculations, we were able to determine an objective level of evidence for all cancer sites, which lends robustness to our results. The large sample size with substantial numbers of cases further enhanced the power of our results and allowed us to perform numerous sub-analyses.

Nevertheless, our umbrella review has some limitations. While similar, each meta-analysis used different sets of search algorithms and inclusion/exclusion criteria. Therefore, merging meta-analyses led to the possibility of artificially introducing heterogeneity and increasing selection and misclassification bias. In addition, the comparability of individual studies is impaired due to different underlying definitions of sedentary behavior and different measures of sedentary behavior in the various studies. Particularly noteworthy is that some of the included meta-analyses contained individual studies whose measure of physical inactivity progressed straight from “walking” to “sitting” and therefore did not adequately capture sedentary behavior [85]. Another aim of this umbrella review was to provide the most up-to-date evidence from the literature. The inclusion of additional individual studies could have introduced bias by different selection criteria. However, the number of newly included individual studies was limited, making relevant bias unlikely. Although the results were consistent across subgroups, residual confounding cannot be ruled out. Furthermore, time spent watching TV tends to be underestimated [86, 87], which is why we only included summary estimates of TV viewing time when no other measurement of exposure was available. In addition, successful alterations of observational associations for modifiable risk factors such as sedentary behavior are not necessarily translated into large preventive benefits for cancer. Although it has been shown that interventions targeting the physical environment effectively reduce sedentary behavior in the majority of populations and settings [88], implementation of randomized controlled trials has been shown to be difficult for behavioral interventions. Finally, it is known that questionnaires generally underestimate the time spent in sedentary behaviors [89]. Since exposure information was mostly based on self-reports and not from objectively assessed methods, this results in potential measurement error. The assessment of total sitting time seems to be most suitable for self-reported sedentary behaviors, which is why we preferred the summary estimate of total sitting time as measurement of sedentary behavior [90]. It is recommended, if possible, to use objective, valid and reliable assessment procedures [89] such as accelerometers in future studies.

## Conclusion

Prolonged sedentary behavior is an independent risk factor for the development of several types of cancer. The strength of the associations varies between individual cancer entities. The association with sedentary behavior is strongest for ovarian, endometrial, breast, colon and rectal cancer incidence. Further studies are needed to determine whether sedentary behavior is an independent risk factor for prostate cancer. For some associations, the effect was diminished when adjusting for obesity. We recommend that all future studies adjust for obesity, as this variable is one of the main confounders between sedentary behavior and cancer. To reduce sedentary behavior, the recommendation of the current World Health Organization Guidelines on physical activity and sedentary behavior [91] should be followed.

## Supplementary Information

Below is the link to the electronic supplementary material.

Supplementary file1 (DOCX 167 KB)

## Data Availability

Available from the authors on request.
