# Supplementary material for: Sedentary behavior and cancer–an umbrella review and meta-analysis
Source: Eur J Epidemiol. 2022 May 25;37(5):447–60. doi: 10.1007/s10654-022-00873-6 (PMC9209390; doi:10.1007/s10654-022-00873-6)
Supplement: Supplementary file 1 — Supplementary file1 (DOCX 167 KB) [file 10654_2022_873_MOESM1_ESM.docx]

Supplementary Index

[Table S1: PubMed search strategy (with limit for systematic reviews and meta-analysis) 2](#_Toc56256307)

[Table S2: PubMed Search strategy (without limit for systematic reviews and meta-analysis) 3](#_Toc56256308)

[Table S3: Included and excluded systematic reviews and meta-analyses, with reasons 4](#_Toc56256309)

[Table S4: Assessing the Methodological Quality of Systematic Reviews – AMSTAR 8](#_Toc56256310)

[Table S5: Summary effect sizes and level of evidence grading of both cohort and case-control studies measuring the association with cancer incidence and mortality 9](#_Toc56256311)

[Table S6: Summary effect sizes and level of evidence grading of cohort studies measuring the association with cancer incidence and mortality 10](#_Toc56256312)

[Table S7: Subgroup analysis of sedentary behavior and ovarian cancer incidence 11](#_Toc56256313)

[Table S8: Subgroup analysis of sedentary behavior and cancer mortality 12](#_Toc56256314)

[Table S9: Subgroup analysis of sedentary behavior and endometrial cancer incidence 13](#_Toc56256315)

[Table S10: Subgroup analysis of sedentary behavior and breast cancer incidence 14](#_Toc56256316)

[Table S11: Subgroup analysis of sedentary behavior and colon cancer incidence 15](#_Toc56256317)

[Table S12: Subgroup analysis of sedentary behavior and rectal cancer incidence 16](#_Toc56256318)

[Table S12: Subgroup analysis of sedentary behavior and rectal cancer incidence 17](#_Toc56256319)

[Table S13: Subgroup analysis of sedentary behavior and prostate cancer incidence 18](#_Toc56256320)

| Table S1: PubMed search strategy (with limit for systematic reviews and meta-analysis) | |
| --- | --- |
| **Set** | **Search Strategy** |
| Limit: Language | (English[lang]) |
| Limit: Exclude animal only | NOT ("Animals"[Mesh] NOT ("Animals"[Mesh] AND "Humans"[Mesh])) |
| Limit: Exclude child only | NOT (("infant"[Mesh] OR "child"[mesh] OR "adolescent"[mh]) NOT (("infant"[Mesh] OR "child"[mesh] OR "adolescent"[mh]) AND "adult"[Mesh])) |
| Limit: Publication Date (Systematic Reviews/Meta-Analyses) | AND ("2000/01/01"[PDAT] : "3000/12/31"[PDAT]) |
| Limit: Publication Type Include Systematic Reviews/Meta-Analyses | AND (systematic[sb] OR meta-analysis[pt] OR “systematic review”[tiab] OR “systematic literature review”[tiab] OR metaanalysis[tiab] OR "meta analysis"[tiab] OR metanalyses[tiab] OR "meta analyses"[tiab] OR "pooled analysis"[tiab] OR “pooled analyses”[tiab] OR "pooled data"[tiab]) |
| Limit: Publication Type Exclude Systematic Reviews/Meta-Analyses | NOT (“comment”[Publication Type] OR “editorial”[Publication Type]) |
| Sedentary | AND (("Sedentary behavior"[mh] OR "Computer time"[tiab] OR "Computer use"[tiab] OR "Screen time"[tiab] OR "Sitting"[tiab] OR "Television"[tiab] OR "TV viewing"[tiab] OR "TV watching"[tiab] OR "Video game"[tiab] OR "Video gaming"[tiab]) OR (("Sedentary"[tiab] OR "Inactivity"[tiab] OR "Physically inactive"[tiab] OR "Sedentarism"[tiab]) NOT medline[sb])) |
| Incidence/Risk / Mortality / Survivor | AND ("risk"[tiab] OR "risks"[tiab] OR "Incidence"[tiab] OR "incident"[tiab] OR "incidents"[tiab] OR "risk"[mh] OR "Incidence"[mh] OR Death[mh] OR Mortalit*[tiab] OR Mortality[mh] OR "Neoplasm Recurrence, Local"[mh] OR Recurrence[mh] OR "Neoplasms, Second Primary"[mh] OR Survival[tiab] OR Death[tiab] OR Dying[tiab] OR Fatal*[tiab] OR Postmortem[tiab] OR Recurrence[tiab] OR "Second cancer"[tiab] OR "Second primary cancer"[tiab] OR "Second neoplasm"[tiab] OR "Second primary neoplasm"[tiab]) |
| Cancer | AND ("Cancer"[tiab] OR "Neoplasm"[tiab] OR "Tumor"[tiab] OR "Carcinogenesis"[tiab] OR "Leukemia"[tiab] OR "Lymphoma"[tiab] OR "Malignancy"[tiab] OR "Blastoma"[tiab] OR "Tumour"[tiab] OR "Melanoma"[tiab] OR "Myeloma"[tiab] OR "Carcinoma"[tiab] OR "Neoplasia"[tiab] OR "Sarcoma"[tiab] OR "Tumors"[tiab] OR "Tumours"[tiab] OR "Neoplasms"[tiab] OR "Adenosarcoma"[tiab] OR "Angiosarcoma"[tiab] OR "Astrocytoma"[tiab] OR "Cholangiocarcinoma"[tiab] OR "Chondrosarcoma"[tiab] OR "Craniopharyngioma"[tiab] OR "Ependymoma"[tiab] OR "Fibrosarcoma"[tiab] OR "Glioma"[tiab] OR "Langerhans Cell Histiocytosis"[tiab] OR "Hodgkin's Disease"[tiab] OR "Leiomyosarcoma"[tiab] OR "Medulloblastoma"[tiab] OR "Mesothelioma"[tiab] OR "Neuroblastoma"[tiab] OR "Rhabdomyosarcoma"[tiab] OR "Osteosarcoma"[tiab]) |

| Table S2: PubMed Search strategy (without limit for systematic reviews and meta-analysis) | |
| --- | --- |
| **Set** | **Search Strategy** |
| Limit: Language | (English[lang]) |
| Limit: Exclude animal only | NOT ("Animals"[Mesh] NOT ("Animals"[Mesh] AND "Humans"[Mesh])) |
| Limit: Exclude child only | NOT (("infant"[Mesh] OR "child"[mesh] OR "adolescent"[mh]) NOT (("infant"[Mesh] OR "child"[mesh] OR "adolescent"[mh]) AND "adult"[Mesh])) |
| Limit: Publication Date | AND ("2015/01/01"[PDAT] : "3000/12/31"[PDAT]) |
| Sedentary | AND (("Sedentary behavior"[mh] OR "Computer time"[tiab] OR "Computer use"[tiab] OR "Screen time"[tiab] OR "Sitting"[tiab] OR "Television"[tiab] OR "TV viewing"[tiab] OR "TV watching"[tiab] OR "Video game"[tiab] OR "Video gaming"[tiab]) OR (("Sedentary"[tiab] OR "Inactivity"[tiab] OR "Physically inactive"[tiab] OR "Sedentarism"[tiab]) NOT medline[sb])) |
| Incidence/Risk / Mortality / Survivor | AND ("risk"[tiab] OR "risks"[tiab] OR "Incidence"[tiab] OR "incident"[tiab] OR "incidents"[tiab] OR "risk"[mh] OR "Incidence"[mh] OR Death[mh] OR Mortalit*[tiab] OR Mortality[mh] OR "Neoplasm Recurrence, Local"[mh] OR Recurrence[mh] OR "Neoplasms, Second Primary"[mh] OR Survival[tiab] OR Death[tiab] OR Dying[tiab] OR Fatal*[tiab] OR Postmortem[tiab] OR Recurrence[tiab] OR "Second cancer"[tiab] OR "Second primary cancer"[tiab] OR "Second neoplasm"[tiab] OR "Second primary neoplasm"[tiab]) |
| Cancer | AND ("Cancer"[tiab] OR "Neoplasm"[tiab] OR "Tumor"[tiab] OR "Carcinogenesis"[tiab] OR "Leukemia"[tiab] OR "Lymphoma"[tiab] OR "Malignancy"[tiab] OR "Blastoma"[tiab] OR "Tumour"[tiab] OR "Melanoma"[tiab] OR "Myeloma"[tiab] OR "Carcinoma"[tiab] OR "Neoplasia"[tiab] OR "Sarcoma"[tiab] OR "Tumors"[tiab] OR "Tumours"[tiab] OR "Neoplasms"[tiab] OR "Adenosarcoma"[tiab] OR "Angiosarcoma"[tiab] OR "Astrocytoma"[tiab] OR "Cholangiocarcinoma"[tiab] OR "Chondrosarcoma"[tiab] OR "Craniopharyngioma"[tiab] OR "Ependymoma"[tiab] OR "Fibrosarcoma"[tiab] OR "Glioma"[tiab] OR "Langerhans Cell Histiocytosis"[tiab] OR "Hodgkin's Disease"[tiab] OR "Leiomyosarcoma"[tiab] OR "Medulloblastoma"[tiab] OR "Mesothelioma"[tiab] OR "Neuroblastoma"[tiab] OR "Rhabdomyosarcoma"[tiab] OR "Osteosarcoma"[tiab]) |

| Table S3: Included and excluded systematic reviews and meta-analyses, with reasons | | | | | | | | | |  |  |  |  |
| --- | --- | --- | --- | --- | --- | --- | --- | --- | --- | --- | --- | --- | --- |
|  |  |  |  | Number of studies | | | | |  |  |  |  |  |
| Author and cancer site | Year | Population (Number. of studies) | Population | Cohort | Case-control | measuring sedentary behavior | Regarding cancer | total | Total population included | Definition of sedentary behavior | Outcome | Selection decision | Reason for exclusion |
| All cancer and cancer mortality |  |  |  |  |  |  |  |  |  |  |  |  |  |
| Schmid et al. [1] | 2014 | North America, Europe, Asia, Australia | General | 21 | 22 | 43 | 43 | 43 | 406,8437 | TV-viewing time | cancer of any type | included |  |
| Ekelund et al. [2] | 2019 | North America (9)†, Europe (3), Asia (1), Australia (1) | General | 14 | 0 | 14 | 13 | 14 | 458,091 (mor.) | daily sitting and TV-viewing time | CVD and cancer mortality | included |  |
| Zhao et al. [3] | 2020 | North America (11), Europe (7), Asia (3), Australia (3) | General | 24 | 0 | 24 | 10 | 24 | 1,156,400 | total sitting time, watching TV, leisure-time sitting | cancer mortality, CVD mortality | included |  |
| Patterson et al. [4] | 2019 | North America (17), Europe (9), Australia (4), Asia (4) | Non-diseased adults in the general population | 34 | 0 | 34 | 19 | 34 | 1,331,468 | total sitting time, occupational sitting, watching TV | all-cause mortality, CVD mortality, diabetes type 2, cancer mortality | included |  |
| Biswas et al. [5] | 2015 | North America (27), Europe (6), Asia (2), Australia (5) | General | 38 | 3 | 41 | 15 | 41 | 744,706 (inc.) | total sitting time, watching TV, leisure-time sitting | all-cause mortality, CVD incidence, CVD mortality, diabetes type 2, cancer incidence, cancer mortality | excluded | same studies as Schmid et al. and Lynch et al. |
| Shen et al. [6] | 2014 | North America (10), Europe (2), Asia (2) | General | 14 | 0 | 14 | 14 | 14 | 857,581 | total sitting time, occupational sitting, watching TV | cancer incidence | excluded | same studies as Schmid et al. |
| Lynch et al. [7] | 2018 | North America (37), Asia (4), Europe (13), Australia (1) | General | 46 | 9 | 55 | 55* | 55 |  | total sitting time, watching TV, leisure-time sitting | cancer incidence, all-cancer mortality | included |  |
| Jochem et al. [8] | 2019 | NA | General | NA | NA | NA | NA | NA | NA | total sitting time, occupational sitting, watching TV | cancer incidence | excluded | narrative review |
| De Rezende et al. [9] | 2014 | NA | General | NA | NA | NA | NA | NA | NA | total sitting time, watching TV, leisure-time sitting | cancer incidence, CVD incidence | excluded | narrative review |
| Thorp et al. [10] | 2011 | NA | General | NA | NA | NA | NA | NA | NA | total sitting time | health outcome, such as Diabetes mellitus type II, cancer and obesity | excluded | narrative review |
| Proper et al. [11] | 2011 | NA | General | NA | NA | NA | NA | NA | NA | total sitting time, watching TV, leisure-time sitting | CVD, endometrial cancer, obesity, waist gain, BMI-gain, Diabetes mellitus type II, all-cause mortality | excluded | narrative review |
| Xu et al. [12] | 2019 | NA | General | NA | NA | NA | NA | NA | NA | total sitting time, watching TV, leisure-time sitting | all-cause mortality | excluded | not measuring cancer |
| Van Uffelen et al. [13] | 2010 | NA | General | NA | NA | NA | NA | NA | NA | prolonged sitting; typically less than 1.5 METs; multiples of the basal metabolic rate | CVD, cancer, diabetes mellitus and, all-cause mortality | excluded | narrative review |
| Lynch et al. [14] | 2010 | NA | General | NA | NA | NA | NA | NA | NA | occupational sitting, watching TV | cancer | excluded | narrative review |
| Lacombe et al. [15] | 2019 | NA | General | NA | NA | NA | NA | NA | NA | sitting time | CVD, cancer, all-cause mortality | excluded | narrative review |
| Katzmarzyk et al. [16] | 2018 | NA | General | NA | NA | NA | NA | NA | NA | total sitting time | all-cause mortality, CVD incidence, CVD mortality, diabetes type 2, cancer incidence, cancer mortality | excluded | narrative review |
| Endometrial cancer |  |  |  |  |  |  |  |  |  |  |  |  |  |
| Moore et al. [17] | 2010 | USA | General | 17 | 0 | 3 | 17 | 17 | NA | total sitting time | endometrial cancer | excluded | same studies as Schmid et al. |
| Breast cancer |  |  |  |  |  |  |  |  |  |  |  |  |  |
| Chong et al. [18] | **2020** | North America (5), Asia (2), Europe (1) | Women | 7 | 0 | 7 | 7 | 7 | 426,506 | total sitting, occupational sitting time, watching TV | breast cancer | included |  |
| Chan et al. [19] | 2019 | North America, Europe, Asia, Australia | Women | 142 | 0 | 6 | 142 | 142 | 8,530,000 | total sitting time, watching TV | pre- and postmenopausal breast cancer | included |  |
| Zhou et al. [20] | 2015 | Europe (9,) China (2), India (1), USA (7), Canada (2) | Women | 7 | 14 | 21 | 21 | 21 | 2,625,772 | total sitting time | breast cancer | included |  |
| Lee et al. [21] | 2021 | Europe (15) Asia (4), USA (9), Canada (3) | Women | 13 | 18 | 31 | 31 | 31 |  | occupational sitting | Breast cancer | excluded | Individual studies were already included or using physical inactivity as reference |
| Ovarian cancer |  |  |  |  |  |  |  |  |  |  |  |  |  |
| Lee, J et al. [22] | 2018 | North America (20), Europe (12), Asia (2) | Women | 17 | 17 | 10 | 34 | 34 | 144,937,608 | total sitting time, occupational sitting time, watching TV | ovarian cancer | excluded | included partially the same population |
| Biller et al. [23] | 2020 | Europe (2), Asia (3), USA (2) | Women | 3 | 4 | 7 | 7 | 7 | 197.416 | Total sitting time, occupational sitting time, watching TV | Ovarian cancer | included |  |
| Colon and rectal cancer | |  |  |  |  |  |  |  |  |  |  |  |  |
| Mahmood et al. [24] | 2017 | North America (17), Europe (14), Asia (6), Australia (1) | General | 17 | 21 | 8 | 38 | 38 | 3,500,500 | sitting time | colon and rectal cancer | included |  |
| Ma et al. [25] | 2017 | North America (10), Europe (12), Asia (1), Australia (2) | General | 17 | 11 | 28 | 28 | 28 | 47,84,339 | total sitting time, occupational sitting time, watching TV | colon and rectal cancer | included |  |
| Cong et al. [26] | 2014 | North America (6), Europe (14), Asia (2), Australia (2) | General | 13 | 11 | 23 | 23 | 23 | 4,324,756 | total sitting time, occupational sitting time, watching TV | colon and rectal cancer | included |  |
| Wang et al. [27] | 2019 | North America (15), Europe (9), Asia (8) | General | 5 | 27 | 3 | 0 | 32 |  | total sitting time | colorectal neoplasia | excluded | not measuring cancer |
| Lee et al. | 2021 | North America (5), Europe (12), Asia (3), Oceania (5) | Working population | 12 | 11 | 23 | 23 | 23 |  | occupational sitting time | Colorectal Cancer | excluded | Individual studies were already included or using physical inactivity as reference |
| Prostate cancer |  |  |  |  |  |  |  |  |  |  |  |  |  |
| Berger et al. [28] | 2019 | Europe (9), USA (2) | General | 12 | 0 | 12 | 12 | 12 | 671,852 | total sitting time | prostate cancer | included |  |
| Post diagnosed sedentary behavior |  |  |  |  |  |  |  |  |  |  |  |  |  |
| Swain et al. [29] | 2020 | North America (22), Europe (2), Australia (5) | Cancer diagnosis | 29 | 16 | 13 | NA | 29 |  | total sitting time, occupational sitting, watching TV | all-cause mortality, colorectal cancer mortality (9) PRO (16) AO (5) | included |  |
| Abbreviations: NA not applicable; PRO Patient-related outcome; AO anthropometric outcome, Inc. incidence, mor. mortality, CVD cardiovascular disease | | | | | | | | | |  |  |  |  |
| *of these, 9 regarding all-cancer mortality and 46 regarding cancer incidence † number of studies regarding the specific region | | | | | | | | |  |  |  |  |  |

| Table S4: Assessing the Methodological Quality of Systematic Reviews – AMSTAR | | | | | | | | | |  | |  | |  | |  | |  | |  | |  | |  |
| --- | --- | --- | --- | --- | --- | --- | --- | --- | --- | --- | --- | --- | --- | --- | --- | --- | --- | --- | --- | --- | --- | --- | --- | --- |
| Author | 1 | 2 | 3 | 4 | 5 | 6 | 7 | 8 | 9 | | 10 | | 11 | | 12 | | 13 | | 14 | | 15 | | 16 | |
| Berger et al. | Yes | Yes | No | Partial yes | Yes | Yes | No | Yes | Yes | | No | | Yes | | Yes | | Yes | | Yes | | Yes | | Yes | |
| Biller et al. | Yes | Yes | No | Partial yes | Yes | Yes | Yes | Yes | Yes | | No | | Yes | | Yes | | Yes | | Yes | | Yes | |  | |
| Chan et al. | Yes | Yes | No | Yes | Yes | No | Yes | Yes | Yes | | No | | Yes | | Yes | | Yes | | Yes | | Yes | | Yes | |
| Cong et al. | Yes | Yes | No | Partial yes | No | Yes | No | Yes | Partial yes | | No | | Yes | | No | | No | | Yes | | Yes | | Yes | |
| Chong et al. | Yes | Yes | No | Partial yes | No | Yes | No | Yes | Partial yes | | No | | Yes | | Yes | | Yes | | Yes | | Yes | | Yes | |
| Ekelund et al. | Yes | Yes | No | Partial yes | Yes | Yes | No | Yes | Partial yes | | No | | Yes | | Yes | | Yes | | Yes | | Yes | | Yes | |
| Lynch et al. | Yes | Yes | No | Partial yes | No | No | No | Partial yes | no | | No | | Yes | | Yes | | Yes | | Yes | | Yes | | No | |
| Ma et al. | Yes | Yes | No | Partial yes | Yes | Yes | No | Yes | Partial yes | | No | | Yes | | Yes | | No | | Yes | | Yes | | Yes | |
| Mahmood et al. | Yes | Yes | No | Partial yes | Yes | Yes | No | Yes | Partial yes | | No | | Yes | | Yes | | Yes | | Yes | | Yes | | Yes | |
| Patterson et al. | Yes | Partial yes | No | Yes | No | Yes | No | Partial yes | Partial yes | | No | | Yes | | No | | Yes | | Yes | | Yes | | Yes | |
| Schmid et al. | Yes | Yes | No | Partial yes | No | Yes | No | Yes | Yes | | No | | Yes | | Yes | | Yes | | Yes | | Yes | | Yes | |
| Swain et al. | Yes | Yes | No | Partial yes | Yes | Yes | Yes | Yes | Yes | | No | | Yes | | Yes | | Yes | | Yes | | Yes | | Yes | |
| Zhao et al. | Yes | Partial yes | No | Partial yes | No | Yes | No | Yes | Partial yes | | No | | Yes | | Yes | | Yes | | Yes | | Yes | | Yes | |
| Zhou et al. | Yes | Partial yes | No | Partial yes | Yes | Yes | No | Partial yes | Partial yes | | No | | Yes | | No | | No | | Yes | | Yes | | Yes | |
| Yes |  |  |  |  |  |  |  |  |  | |  | |  | |  | |  | |  | |  | |  | |
| Partial yes |  |  |  |  |  |  |  |  |  | |  | |  | |  | |  | |  | |  | |  | |
| No |  |  |  |  |  |  |  |  |  | |  | |  | |  | |  | |  | |  | |  | |
| 1. Did the research questions and inclusion criteria for the review include the components of PICO? 2. Did the report of the review contain an explicit statement that the review methods were established prior to the conduct of the review and did the report justify any significant deviations from the protocol 3. Did the review authors explain their selection of the study designs for inclusion in the review? 4. Did the review authors use a comprehensive literature search strategy? 5. Did the review authors perform study selection in duplicate? 6. Did the review authors perform data extraction in duplicate? 7. Did the review authors provide a list of excluded studies and justify the exclusions? 8. Did the review authors describe the included studies in adequate detail? 9. Did the review authors use a satisfactory technique for assessing the risk of bias (RoB) in individual studies that were included in the review? 10. Did the review authors report on the sources of funding for the studies included in the review? 11. If meta-analysis was performed did the review authors use appropriate methods for statistical combination of results? 12. If meta-analysis was performed, did the review authors assess the potential impact of RoB in individual studies on the results of the meta-analysis or other evidence synthesis? 13. Did the review authors account for RoB in individual studies when interpreting/ discussing the results of the review? 14. Did the review authors provide a satisfactory explanation for, and discussion of, any heterogeneity observed in the results of the review? 15. If they performed quantitative synthesis did the review authors carry out an adequate investigation of publication bias (small study bias) and discuss its likely impact on the results of the review? 16. Did the review authors report any potential sources of conflict of interest, including any funding they received for conducting the review? | | | | | | | | | | | | | | | | | | | | | | | |  |

| Table S5: Summary effect sizes and level of evidence grading of both cohort and case-control studies measuring the association with cancer incidence and mortality | | | | | | | | | | | | |
| --- | --- | --- | --- | --- | --- | --- | --- | --- | --- | --- | --- | --- |
|  |  |  |  |  |  |  |  |  |  |  | **Excess significance** | |
| **Cancer** | **Number of effect sizes** | **Sedentary behavior** | **Number of cases/sample size** | **Largest study (smallest SE)** | **PI excluding the null** | **Random effects, RR 95%CI** | ***p-v*alue (REM)** | **I², 95%CI** | **Egger´s P** | **Trim and Fill, RR (95%CI) +AS** | **O/E** | **p-value*** |
| *Highly suggestive level of evidence* |  |  |  |  |  |  |  |  |  |  |  |  |
| All-cancer mortality | 17 (17 cohort) | TS, OS, TV | 50406/683321 | 1.11 (1.05, 1.17) | Yes | 1.18 (1.09-1.26) | <10^-6^ | 57 (28-74) | <0.01 | 1.11 (1.03-1.20) +7 | 8/7.35 | 0.643 |
| *Suggestive level of evidence* |  |  |  |  |  |  |  |  |  |  |  |  |
| Colon cancer | 34 (21 cohort, 13 case-control) | TS, OS, TV | 30688/3765814 | 1.03 (0.96, 1.10) | No | 1.25 (1.16-1.33) | <10^-6^ | 51 (31-68) | 0.02 | 1.16 (1.07-1.25) +10 | 11/2.52 | 0.00002 |
| Rectal cancer | 28 (15 cohort, 13 case-control) | TS, OS, TV | 18411/3138440 | 1.10 (1.00, 1.20) | Yes | 1.07 (1.01-1.12) | 0.001 | 12 (0-44) | 0.72 | No missing studies | 3/6.14 | Not pertinent |
| Breast cancer | 29 (15 cohort, 14 case-control) | TS, OS, TV | 98451/2741926 | 1.00 (0.92, 1.09) | No | 1.08 (1.04-1.11) | <10^-6^ | 24 (0-50) | 0.11 | 1.08 (1.05-1.22) +1 | 5/1.45 | 0.0003 |
| Endometrial cancer | 11 (6 cohort, 5 case-control) | RS,TV | 4561/281530 | 1.23 (0.96, 1.57) | Yes | 1.29 (1.16-1.45) | <10^-6^ | 14 (0-55) | 0.15 | 1.30 (1.17-1.46) +2 | 4/4.97 | Not pertinent |
| Ovarian cancer | 8 (4 cohort, 4 case-control) | TS, OS, TV | 1463/200424 | 1.44 (1.12, 1.85) | No | 1.29 (1.08-1.56) | <10^-3^ | 26 (0-67) | 0.85 | 1.28 (1.07-1.52) +1 | 3/4.66 | Not pertinent |
| *Weak level of evidence* |  |  |  |  |  |  |  |  |  |  |  |  |
| Prostate cancer | 14 (13 cohort, 1 case-control) | TS, OS, TV | 31351/593049 | 0.97 (0.91, 1.03) | No | 1.08 (1.00-1.17) | 0.048 | 55 (19-75) | 0.18 | 0.99 (0.91-1.09) +6 | 3/1.59 | 0.207 |
| *Not statistically significant associations* | |  |  |  |  |  |  |  |  |  |  |  |
| Lung cancer | 9 (8 cohort, 1 case-control) | TS, OS, TV | 12460/1006535 | 1.00 (0.90-1.11) | No | 1.07 (0.91-1.25) | >0.05 | 70 (45-84) | 0.14 | NP | NP | NP |
| Gastric cancer | 8 (6 cohort, 2 case-control) | TS, OV, TV | 2676/306996 | 0.94 (0.41-2.91) | No | 1.07 (0.94-1.23) | >0.05 | 0 (0-55) | 0.98 | NP | NP | NP |
| Esophageal cancer | 7 (6 cohort, 1 case-control) | TS, TV, OS | 1558/842105 | 1.14 (0.87-1.49) | No | 1.06 (0.91-1.22) | >0.05 | 0 (0-54) | 0.72 | NP | NP | NP |
| Testicular cancer | 3 (3 case-control) | TV, OS | 1375/6963 | 1.71 (1.08-2.72) | No | 1.27 (0.84-1.29) | >0.05 | 48 (0-85) | NP | NP | NP | NP |
| renal cell cancer | 3 (3 cohort) | TS, TV | 1839/469541 | 1.08 (0.92-1.27) | No | 1.08 (0.95-1.32) | >0.05 | 0 (0-10) | NP | NP | NP | NP |
| Non-Hodgkin lymphoma | 3 (2 cohort, 1 case-control) | TS, TV | 4886/308402 | 0.95 (0.78-1.15) | No | 1.05 (0.94-1.17) | >0.05 | 0 (0-68) | NP | NP | NP | NP |
| Gallbladder cancer | 2 (2 cohort) | TV, OS | 105/180025 | 0.56 (0.27-1.17) | NA | 1.10 (0.57-2.15) | >0.05 | 49 (0-83) | NP | NP | NP | NP |
| Head and Neck cancer | 1 (1 cohort) | TV | 371/146722 | 1.28 (0.87-1.70) | NA | 1.28 (0.87-1.70) | >0.05 | NP | NP | NP | NP | NP |
| Liver cancer | 3 (3 cohort) | TV | 548/249781 | 1.20 (0.81-1.77) | No | 1.04 (0.77-1.40) | >0.05 | 34 (0-75) | NP | NP | NP | NP |
| Melanoma | 2 (2 cohort) | TV | 1154/146722 | 1.03 (0.90-1.18) | NA | 1.03 (0.93-1.15) | >0.05 | NP | NP | NP | NP | NP |
| Multiple Myeloma | 1 (1 cohort) | TV | 414/146722 | 1.27 (0.78-2.07) | NA | 1.27 (0.78-2.07) | >0.05 | NP | NP | NP | NP | NP |
| Pancreatic cancer | 2 (2 cohort) | TV, OS | 448/166722 | 1.09 (0.89-1.34) | NA | 1.16 (0.95-1.40) | >0.05 | 36 (0-77) | NP | NP | NP | NP |
| **Abbreviations:** TS total sitting, OS occupational sitting, TV Viewing time, RS recreational sitting RR relative risk, CI confidence interval, REM random effect model, PI prediction interval, O/E number of observed/expected studies, Not pertinent number of observed studies was smaller than the number of expected studies, NP not performed, NA not applicable AS added studies, SE Standard error *p-value of the excess significance test | | | | | | | | | | | | |

| Table S6: Summary effect sizes and level of evidence grading of cohort studies measuring the association with cancer incidence and mortality | | | | | | | | | | | | |
| --- | --- | --- | --- | --- | --- | --- | --- | --- | --- | --- | --- | --- |
|  |  |  |  |  |  |  |  |  |  |  | **Excess significance** | |
| **Cancer** | **Number of effect sizes** | **Sedentary behavior** | **Number of cases/sample size** | **Largest study (smallest SE)** | **PI excluding the null** | **Random effect, RR 95%CI** | ***p-va*lue (REM)** | **I², 95%CI** | **Egger´s P** | **Trim and Fill, RR (95%CI) +AS** | **O/E** | **p-value*** |
| *Convincing level of evidence* |  |  |  |  |  |  |  |  |  |  |  |  |
|  |  |  |  |  |  |  |  |  |  |  |  |  |
| *Highly suggestive level of evidence* |  |  |  |  |  |  |  |  |  |  |  |  |
| All-cancer mortality | 17 | TS, OS, TV | 50406/683321 | 1.11 (1.05, 1.17) | Yes | 1.18 (1.09-1.26) | <10^-6^ | 57 (28-74) | <0.01 | 1.11 (1.03-1.2) +7 | 8/7.35 | 0.643 |
| *Suggestive level of evidence* |  |  |  |  |  |  |  |  |  |  |  |  |
| Colon cancer | 21 | TS, OS | 27754/3745384 | 1.03 (0.96, 1.10) | No | 1.20 (1.10-1.28) | <10^-6^ | 57 (31-74) | 0.88 | 1.15 (1.04-1.28) +3 | 6/1.79 | 0.007 |
| Breast cancer | 15 | TS, TV,OS | 78465/2686362 | 1.00 (0.92, 1.09) | No | 1.07 (1.04-1.11) | 0.0001 | 15 (0-50) | 0.06 | 1.07 (1.03-1.10) +3 | 2/0.70 | 0036 |
| Endometrial cancer | 6 | TS, RS | 2273/272472 | 1,80 (1,15-2,83) | Yes | 1.35 (1.16- 1.55) | <10^-6^ | 16 (0-71) | 0.26 | 1.37 (1.03-1.10) +1 | 3/2.85 | 1 |
| *Weak level of evidence* |  |  |  |  |  |  |  |  |  |  |  |  |
| Ovarian cancer | 4 | TV,OS | 1164/208254 | 1.44 (1.12, 1.85) | No | 1.29 (1.00-1.66) | 0.049 | 11 (0-86) | 0.51 | 1.25 (1.00-1.56) +1 | 1/2.40 | Not pertinent |
| Rectal cancer | 15 | TS,OS | 16411/3200227 | 1.10 (1.00, 1.20) | No | 1.07 (1.02-1.13) | 0.012 | 0 (0-31) | 0.74 | 1.08 (1.03- 1.14) +2 | 2/4.91 | Not pertinent |
| Prostate cancer | 13 | TS, OS, TV | 31322/587034 | 0.97 (0.91, 1.03) | No | 1.08 (1.00-1.17) | 0.0455 | 59 (24-78) | 0.11 | 0.99 (0.91-1.09) +5 | 3/1.59 | 0.207 |
| *Not statistically significant associations* |  |  |  |  |  |  |  |  |  |  |  |  |
| Lung cancer | 8 | TS, OS, TV | 12459/1004753 | 1.00 (0.90-1.11) | No | 1.05 (0.86-1.24) | >0.05 | 72 (46-85) | NP | NP | NP | NP |
| Gastric cancer | 6 | TS, TV, OS | 2383/720877 | 0.94 (0.41-2.91) | No | 1.09 (095-1.26) | >0.05 | 0 (0-50) | NP | NP | NP | NP |
| Esophageal cancer | 6 | TS, TV, OS | 1508/842050 | 1.14 (0.87-1.49) | No | 1.06 (0.91-1.22) | >0.05 | 0 (0-54) | NP | NP | NP | NP |
| Renal cell cancer | 3 | TS, TV | 1839/469541 | 1.08 (0.92-1.27) | No | 1.08 (0.95-1.32) | >0.05 | 0 (0-10) | NP | NP | NP | NP |
| Non-Hodgkin lymphoma | 2 | TS, TV | 3867/303402 | 0.95 (0.78-1.15) | NA | 1.08 (0.93-1.25) | >0.05 | 18.5 (0-87) | NP | NP | NP | NP |
| Gallbladder cancer | 2 | TV, OS | 105/180025 | 0.56 (0.27-1.17) | NA | 1.10 (0.57-2.15) | >0.05 | 49 (0-83) | NP | NP | NP | NP |
| Head and Neck cancer | 1 | TV | 371/146722 | 1.28 (0.87-1.70) | NA | 1.28 (0.87-1.70) | >0.05 | NP | NP | NP | NP | NP |
| Liver cancer | 3 | TV, OS | 548/249781 | 1.20 (0.81-1.77) | No | 1.04 (0.77-1.40) | >0.05 | 34 (0-75) | NP | NP | NP | NP |
| Melanoma | 2 | TV | 1154/146722 | 1.03 (0.90-1.18) | NA | 1.03 (0.93-1.15) | >0.05 | NP | NP | NP | NP | NP |
| Multiple Myeloma | 1 | TV | 414/146722 | 1.27 (0.78-2.07) | NA | 1.27 (0.78-2.07) | >0.05 | NP | NP | NP | NP | NP |
| Pancreatic cancer | 2 | TV, OS | 448/166722 | 1.09 (0.89-1.34) | No | 1.16 (0.95-1.40) | >0.05 | 36 (0-77) | NP | NP | NP | NP |
| **Abbreviations**: TS total sitting, OS occupational sitting, TV Viewing time, RS recreational sitting RR relative risk, CI confidence interval, REM random effect model, PI prediction interval, O/E number of observed/expected studies, Not pertinent number of observed studies was smaller than the number of expected studies, NP not performed, NA not applicable AS added studies, SE Standard error | | | | | | | | | | | | |
| *p-value of the excess significance test |  |  |  |  |  |  |  |  |  |  |  |  |

| Table S7: Subgroup analysis of sedentary behavior and ovarian cancer incidence | | | | | | | | |
| --- | --- | --- | --- | --- | --- | --- | --- | --- |
|  |  | **Largest study** | | **REML** | |  |  |  |
| **Variable** | **Number of included RR** | **RR** | **95% CI** | **RR (high vs low SB)** | **95 % CI** | **I² (%)** | **Q (diff)** | **p (diff)*** |
| **Region** |  |  |  |  |  |  |  |  |
| USA | 2 | 1.77 | 1.01-3.12 | 1.24 | 0.92-1.67 | 62% |  |  |
| Europe | 2 | 1.80 | 1.02-3.17 | 1.02 | 0.24-4.26 | 69% |  |  |
| Asia | 4 | 1.07 | 0.77-1.48 | 1.36 | 0.95-1.94 | 4% | 0.25 | **0.88** |
| **Study design** |  |  |  |  |  |  |  |  |
| Case-control | 4 | 1.77 | 1.01-3.12 | 1.33 | 0.92-1.93 | 51% |  |  |
| Cohort | 4 | 1.80 | 1.02-3.17 | 1.29 | 1.00-1.66 | 11% | 0.02 | **.89** |
| **Adjusted for BMI** |  | . |  |  |  |  |  |  |
| No | 3 | 1.80 | 1.02-3.17 | 1.18 | 0.71-1.94 | 58% |  |  |
| Yes | 5 | 1.77 | 1.01-3.12 | 1.36 | 1.10-1.66 | 0% | 0.27 | **.60** |
| **Adjusted for menopausal status** |  |  |  |  |  |  |  |  |
| No | 3 | 1.80 | 1.02-3.17 | 1.18 | 0.71-1.94 | 38% |  |  |
| Yes | 5 | 1.77 | 1.01-3.12 | 1.36 | 1.10-1.66 | 0% | 0.27 | **.60** |
| **Adjusted for smoking** |  |  |  |  |  |  |  |  |
| No | 1 | 1.80 | 1.04-3.10 | 1.80 | 1.04-3.10 | 0% |  |  |
| Yes | 7 | 1.77 | 1.01-3.12 | 1.25 | 1.03-1.51 | 23% | 1.54 | **.21** |
| **Adjusted for alcohol consumption** |  |  |  |  |  |  |  |  |
| No | 5 | 1.80 | 1.02-3.17 | 1.23 | 1.00-1.51 | 45% |  |  |
| Yes | 3 | 1.77 | 1.01-3.12 | 1.74 | 1.12-2.69 | 0% | 2.00 | **.16** |
| **Adjusted for oral contraception** |  |  |  |  |  |  |  |  |
| No | 2 | 1.80 | 1.02-3.17 | 1.20 | 0.24-4.26 | 69% |  |  |
| Yes | 6 | 1.77 | 1.01-3.12 | 1.27 | 1.05-1.54 | 13% | 0.09 | **.76** |
| **Adjusted for family history of cancer** |  |  |  |  |  |  |  |  |
| No | 4 | 1.80 | 1.02-3.17 | 1.46 | 1.17-1.81 | 7% |  |  |
| Yes | 4 | 1.77 | 1.01-3.12 | 1.16 | 0.96-1.40 | 21% | 2.43 | **.12** |
| **Sedentary behavior** |  |  |  |  |  |  |  |  |
| Occupational sitting | 2 | 1.51 | 0.48-4.72 | 0.87 | 0.24-3.13 | 45% |  |  |
| Total sitting | 2 | 1.77 | 1.01-3.12 | 1.30 | 0.80-2.11 | 57% |  |  |
| Tv-viewing time | 4 | 1.80 | 1.02-3.17 | 1.36 | 1.05-1.75 | 36% | 0.45 | **.80** |
| **Abbreviations:** RR relative risk, CI confidence interval, REML random effect model, BMI Body-Mass-Index | | | | |  |  |  |  |
| *statistical difference in the subgroups |  |  |  |  |  |  |  |  |

| Table S8: Subgroup analysis of sedentary behavior and all-cancer mortality | | | | | |  |  |  |
| --- | --- | --- | --- | --- | --- | --- | --- | --- |
|  |  | **Largest study** | | **REML** | |  |  |  |
| **Variable** | **Number of included RR** | **RR** | **95% CI** | **RR (high vs low SB)** | **95% CI** | **I² (%)** | **Q (diff)** | **p (diff)*** |
| **Region** |  |  |  |  |  |  |  |  |
| Asia | 1 | 1.77 | 1.01-3.12 | 1.26 | 1.13-1.41 | NA |  |  |
| Australia | 1 | 1.80 | 1.02-3.17 | 1.48 | 0.88-2.49 | NA |  |  |
| North America | 15 | 1.99 | 1.25-3.17 | 1.16 | 1.08-1.25 | 9% | 2.10 | **0.34** |
| **Adjusted for BMI** |  |  |  |  |  |  |  |  |
| No | 8 | 1.99 | 1.25-3.17 | 1.22 | 1.09-1.36 | 70% |  |  |
| Yes | 9 | 1.62 | 1.07-2.45 | 1.14 | 1.04-1.34 | 57% | 0.97 | **.32** |
| **Adjusted for smoking** |  |  |  |  |  |  |  |  |
| No | 9 | 1.73 | 1.11-2.71 | 1.24 | 1.14-1.34 | 0% |  |  |
| Yes | 8 | 1.99 | 1.25-3.17 | 1.17 | 1.08-1.28 | 58% | 0.75 | **.38** |
| **Adjusted for alcohol consumption** |  |  |  |  |  |  |  |  |
| No | 6 | 1.73 | 1.11-2.71 | 1.20 | 1.13-1.28 | 11% |  |  |
| Yes | 4 | 1.99 | 1.25-3.17 | 1.16 | 1.00-1.34 | 66% | 0.47 | **.67** |
| **Adjusted for family history of cancer** |  |  |  |  |  |  |  |  |
| No | 16 | 1.99 | 1.25-3.17 | 1.20 | 1.11-1.24 | 59% |  |  |
| Yes | 1 | 1.04 | 0.98-1.10 | 1.04 | 0.98-1.10 | 0% | 8.40 | **.003** |
| **Sedentary behavior** |  |  |  |  |  |  |  |  |
| Occupational sitting | 1 | 1.11 | 1.05-1.17 | 1.11 | 1.05-1.17 | NA |  |  |
| Total sitting | 6 | 1.52 | 1.01-2.27 | 1.12 | 0.98-1.27 | 61% |  |  |
| Tv-viewing time | 10 | 1.99 | 1.25-3.17 | 1.26 | 1.12-1.41 | 67% | 3.90 | **.11** |
| **Abbreviations:** RR relative risk, CI confidence interval, REML random effect model, BMI Body-Mass-Index, NA not applicable | | | | | | |  |  |
| *statistical difference in the subgroups | |  |  |  |  |  |  |  |

| Table S9: Subgroup analysis of sedentary behavior and endometrial cancer incidence | | | | | | | |  |
| --- | --- | --- | --- | --- | --- | --- | --- | --- |
|  |  | **Largest study** | | **REML** | |  |  |  |
| **Variable** | **Number of included RR** | **RR** | **95% CI** | **RR (high vs low SB)** | **95% CI** | **I² (%)** | **Q (diff)** | **p (diff)*** |
| **Region** |  |  |  |  |  |  |  |  |
| North America | 4 | 1.52 | 1.07-2.16 | 1.33 | 1.15-1.45 | 0% |  |  |
| Europe | 3 | 1.80 | 1.14-2.83 | 1.52 | 1.20-1.93 | 0% |  |  |
| Asia | 4 | 1.20 | 0.70-2.00 | 0.97 | 0.75-1.26 | 0% | 6.70 | **0.035** |
| **Study design** |  |  |  |  |  |  |  |  |
| Case-control | 5 | 1.52 | 1.07-2.16 | 1.20 | 0.96-1.50 | 16% |  |  |
| Cohort | 6 | 1.80 | 1.14-2.83 | 1.35 | 1.17-1.56 | 16% | 0.69 | **.4** |
| **Adjusted for BMI** |  |  |  |  |  |  |  |  |
| No | 6 | 1.80 | 1.14-2.83 | 1.40 | 1.20-1.64 | 0% |  |  |
| Yes | 5 | 1.52 | 1.07-2.16 | 1.15 | 0.91-1.44 | 38% | 2.07 | **.15** |
| **Adjusted for smoking** |  |  |  |  |  |  |  |  |
| No | 4 | 1.80 | 1.14-2.83 | 1.24 | 0.95-1.62 | 45% |  |  |
| Yes | 7 | 1.52 | 1.07-2.16 | 1.33 | 1.16-1.53 | 0% | 0.20 | **.65** |
| **Adjusted for alcohol consumption** |  |  |  |  |  |  |  |  |
| No | 9 | 1.80 | 1.14-2.83 | 1.31 | 1.17-1.45 | 5% |  |  |
| Yes | 2 | 1.05 | 0.51-2.15 | 0.82 | 0.41-1.65 | 20% | 1.86 | **.019** |
| **Adjusted for family history of cancer** |  |  |  |  |  |  |  |  |
| No | 9 | 1.80 | 1.14-2.83 | 1.29 | 1.15-1.44 | NA | NA | **NA** |
| Yes | 0 |  |  |  |  |  |  |  |
| **Adjusted for hormone status** |  |  |  |  |  |  |  |  |
| No | 5 | 1.80 | 1.14-2.83 | 1.22 | 0.94-1.60 | 35% |  |  |
| Yes | 6 | 1.52 | 1.07-2.16 | 1.33 | 1.16-1.53 | 0% | 0.432 | **.57** |
| **Adjusted for menopausal status** |  |  |  |  |  |  |  |  |
| No | 4 | 1.80 | 1.14-2.83 | 1.37 | 1.09-1.73 | 0% |  |  |
| Yes | 7 | 1.52 | 1.07-2.16 | 1.26 | 1.09-1.46 | 31% | 0.38 | **.54** |
| **Sedentary behavior** |  |  |  |  |  |  |  |  |
| Occupational sitting | 5 | 1.29 | 0.92-1.79 | 1.07 | 0.85-1.35 | 9% |  |  |
| Total sitting | 3 | 1.80 | 1.14-2.83 | 1.36 | 1.16-1.60 | 0% |  |  |
| Recreational sitting | 2 | 1.52 | 1.07-2.16 | 1.51 | 1.18-1.94 | 0% |  |  |
| TV-viewing time | 1 | 1.05 | 0.51-2.15 | 1.05 | 0.51-2.15 | NA | 4.71 | **.19** |
| **Abbreviations**: RR relative risk, CI confidence interval, REML random effect model, BMI Body-Mass-Index, NA not applicable | | | | | | |  |  |
| *statistical difference in the subgroups |  |  |  |  |  |  |  |  |

| Table S10: Subgroup analysis of sedentary behavior and breast cancer incidence | | | | | |  |  |  |
| --- | --- | --- | --- | --- | --- | --- | --- | --- |
|  |  | **Largest study** | | **REML** | |  |  |  |
| **Variable** | **Number of included RR** | **RR** | **95% CI** | **RR (high vs low SB)** | **95% CI** | **I² (%)** | **Q (diff)** | **p (diff)*** |
| **Region** |  |  |  |  |  |  |  |  |
| North America | 18 | 1.56 | 0.78-3.13 | 1.06 | 1.03-1.13 | 23% |  |  |
| Europe | 15 | 2.08 | 1.09-4.01 | 1.09 | 1.03-1.16 | 34% |  |  |
| Asia | 5 | 1.45 | 0.92-2.32 | 1.15 | 0.98-1.37 | 0,0% | 1.32 | **0.52** |
| **Study design** |  |  |  |  |  |  |  |  |
| Case-control | 18 | 1.85 | 0.99-3.47 | 1.09 | 1.03-1.16 | 15% |  |  |
| Cohort | 20 | 2.08 | 1.09-4.01 | 1.07 | 1.03-1.11 | 22% | 0.012 | **.91** |
| **Menopausal status** |  |  |  |  |  |  |  |  |
| ND | 20 | 2.08 | 1.09-4.01 | 1.09 | 1.05-1.14 | 4% |  |  |
| Postmenopausal | 8 | 1.52 | 1.02-2.27 | 1.03 | 0.89-1.18 | 52% |  |  |
| Premenopausal | 6 | 1.56 | 0.78-3.13 | 0.99 | 0.88-1.12 | 17% | 2.62 | **.27** |
| **Adjusted for BMI** |  |  |  |  |  |  |  |  |
| No | 20 | 2.08 | 1.09-4.01 | 1.09 | 1.03-1.15 | 28% |  |  |
| Yes | 18 | 1.52 | 1.02-2.27 | 1.07 | 1.02-1.15 | 6% | 0.02 | **.88** |
| **Adjusted for hormonal status** |  |  |  |  |  |  |  |  |
| No | 7 | 2.08 | 1.09-4.01 | 1.06 | 0.83-1.37 | 57% |  |  |
| Yes | 27 | 1.85 | 0.99-3.47 | 1.07 | 1.04-1.13 | 12% | 0.0046 | **.95** |
| **Adjusted for smoking** |  |  |  |  |  |  |  |  |
| No | 18 | 2.08 | 1.09-4.01 | 1.09 | 1.03-1.16 | 7% |  |  |
| Yes | 16 | 1,45 | 0.92-2.32 | 1.06 | 1.01-1.11 | 38% | 0.49 | **.48** |
| **Adjusted for alcohol consumption** |  |  |  |  |  |  |  |  |
| No | 21 | 2.08 | 1.09-4.01 | 1.05 | 1.04-1.17 | 28% |  |  |
| Yes | 13 | 1.45 | 0.92-2.32 | 1.06 | 1.01-1.16 | 20% | 1.00 | **.31** |
| **Adjusted for family history of cancer** |  |  |  |  |  |  |  |  |
| No | 18 | 2.08 | 1.09-4.01 | 1.08 | 1.03-1.13 | 0% |  |  |
| Yes | 16 | 1.85 | 0.99-3.47 | 1.09 | 1.02-1.17 | 46% | 0.12 | **.72** |
| **Sedentary behavior** |  |  |  |  |  |  |  |  |
| Occupational sitting time | 20 | 2.08 | 1.09-4.01 | 1.08 | 1.03-1.12 | 19% |  |  |
| Total sitting time | 9 | 1.52 | 1.02-2.27 | 1.13 | 1.01-1.27 | 44% |  |  |
| Tv-viewing time | 5 | 1.45 | 0.92-2.32 | 1.09 | 0.96-1.23 | 30% | 0.65 | **.72** |
| **Abbreviations:** RR relative risk, CI confidence interval, REML random effect model, BMI Body-Mass-Index | | | | |  |  |  |  |
| *statistical difference in the subgroups | |  |  |  |  |  |  |  |

| Table S11: Subgroup analysis of sedentary behavior and colon cancer incidence | | | | | |  |  |  |
| --- | --- | --- | --- | --- | --- | --- | --- | --- |
|  |  | **Largest study** | | **REML** | |  |  |  |
| **Variable** | **Number of included RRs** | **RR** | **95% CI** | **RR (high vs low SB)** | **95% CI** | **I² (%)** | **Q (diff)** | **p (diff)*** |
| **Region** |  |  |  |  |  |  |  |  |
| North America | 8 | 1.80 | 1.60-2.20 | 1.43 | 1.21-1.70 | 57% |  |  |
| Europe | 18 | 1.67 | 0.89-3.12 | 1.22 | 1.16-1.23 | 3% |  |  |
| Asia | 7 | 5.26 | 1.30-20.00 | 1.05 | 0.98-1.14 | 40% |  |  |
| Australia | 1 | 1.48 | 0.86-2.56 | 1.48 | 0.86-2.56 | NA | 20.00 | **0.0001** |
| **Study design** |  |  |  |  |  |  |  |  |
| Case-control | 13 | 5.26 | 1.30-20.00 | 1.48 | 1.26-1.74 | 17% |  |  |
| Cohort | 21 | 1.80 | 1.60-2.20 | 1.20 | 1.12-1.27 | 58% | 5.40 | **.019** |
| **Adjusted for BMI** |  |  |  |  |  |  |  |  |
| No | 19 | 5.26 | 1.30-20.00 | 1.30 | 1.17-1.44 | 69% |  |  |
| Yes | 15 | 1.67 | 0.89-3.12 | 1.19 | 1.09-1.30 | 0% | 1.44 | **.23** |
| **Adjusted for smoking** |  |  |  |  |  |  |  |  |
| No | 18 | 2.14 | 1.05-4.35 | 1.28 | 1.16-1.42 | 69% |  |  |
| Yes | 16 | 5.26 | 1.30-20.00 | 1.20 | 1.11-1.30 | 11% | 0.95 | **.33** |
| **Adjusted for alcohol consumption** |  |  |  |  |  |  |  |  |
| No | 20 | 2.14 | 1.05-4.35 | 1.28 | 1.16-1.42 | 65% |  |  |
| Yes | 14 | 5.26 | 1.30-20.00 | 1.22 | 1.12-1.33 | 16% | 0.52 | **.47** |
| **Adjusted for family history of cancer** |  |  |  |  |  |  |  |  |
| No | 28 | 5.26 | 1.30-20.00 | 1.25 | 1.15-1.36 | 60% |  |  |
| Yes | 6 | 1.39 | 1.12-1.72 | 1.25 | 1.13-1.39 | 0% | 0.01 | **.99** |
| **Sex** |  |  |  |  |  |  |  |  |
| both | 5 | 1.67 | 0.89-3.12 | 1.34 | 1.1-1.62 | 0% |  |  |
| men | 16 | 5.26 | 1.30-20.00 | 1.25 | 1.13-1.38 | 38% |  |  |
| women | 13 | 2.14 | 1.05-4.35 | 1.21 | 1.09-1.36 | 0% | 0.72 | **.70** |
| **Sedentary behavior** |  |  |  |  |  |  |  |  |
| Occupational sitting | 23 | 1.80 | 1.60-2.20 | 1.22 | 1.12-1.32 | 59% |  |  |
| Total sitting | 9 | 5.26 | 1.30-20.00 | 1.37 | 1.16-1.6 | 38% |  |  |
| TV-viewing time | 2 | 1.47 | 0.85-2.54 | 1.27 | 1.01-1.58 | 0% | 1.67 | **.43** |
| **Abbreviations:** RR relative risk, CI confidence interval, REML random effect model, BMI Body-Mass-Index, NA not applicable | | | | | | |  |  |
| *statistical difference in the subgroups |  |  |  |  |  |  |  |  |

| Table S12: Subgroup analysis of sedentary behavior and rectal cancer incidence | | | | | |  |  |  |
| --- | --- | --- | --- | --- | --- | --- | --- | --- |
|  |  | **Largest study** | | **REML** | |  |  |  |
| **Variable** | **Number of included RRs** | **RR** | **95% CI** | **RR (high vs low SB)** | **95% CI** | **I² (%)** | **Q (diff)** | **p (diff)*** |
| **Region** |  |  |  |  |  |  |  |  |
| North America | 6 | 1.52 | 0.97-2.38 | 1.24 | 0.97-1.56 | 32% |  |  |
| Europe | 15 | 1.41 | 0.73-2.78 | 1.06 | 1.01-1.12 | 6% |  |  |
| Asia | 6 | 2.27 | 0.67-7.69 | 0.95 | 0.73-1.24 | 0% |  |  |
| Australia | 1 | 1.44 | 0.96-2.18 | 1.44 | 0.96-2.18 | NA | 4.10 | **0.24** |
| **Study design** |  |  |  |  |  |  |  |  |
| Case-control | 13 | 2.27 | 0.67-7.69 | 1.06 | 0.85-1.32 | 42% |  |  |
| Cohort | 15 | 1.41 | 0.73-2.78 | 1.07 | 1.02-1.13 | 0% | 0.02 | **.93** |
| **Sex** |  |  |  |  |  |  |  |  |
| both | 5 | 2.44 | 1.03-5.78 | 1.05 | 0.69-1.65 | 54% |  |  |
| men | 13 | 2.27 | 0.67-7.69 | 1.08 | 1.02-1.14 | 0% |  |  |
| women | 10 | 1.48 | 0.87-2.42 | 1.00 | 0.88-1.13 | 0% | 1.22 | **.54** |
| **Adjusted for BMI** |  |  |  |  |  |  |  |  |
| No | 16 | 2.27 | 0.67-7.69 | 1.07 | 1.01-1.13 | 30% |  |  |
| Yes | 12 | 1.44 | 0.96-2.18 | 1.06 | 0.94-1.19 | 0% | 0.03 | **.85** |
| **Adjusted for smoking** |  |  |  |  |  |  |  |  |
| No | 16 | 1.52 | 0.97-2.38 | 1.07 | 1.01-1.13 | 24% |  |  |
| Yes | 12 | 2.27 | 0.67-7.69 | 1.07 | 0.94-1.23 | 0% | 0.01 | **.94** |
| **Adjusted for alcohol consumption** |  |  |  |  |  |  |  |  |
| No | 18 | 1.52 | 0.97-2.38 | 1.08 | 1.02-1.13 | 21% |  |  |
| Yes | 10 | 2.27 | 0.67-7.69 | 1,02 | 0.88-1.18 | 0% | 0.42 | **.51** |
| **Adjusted for family history of cancer** |  |  |  |  |  |  |  |  |
| No | 24 | 2.27 | 0.67-7.69 | 1.07 | 1.02-1.13 | 11% |  |  |
| Yes | 4 | 1.09 | 0.76-1.55 | 1.02 | 0.83-1.24 | 37% | 0.24 | **.62** |
| **Sedentary behavior** |  |  |  |  |  |  |  |  |
| Occupational sitting | 18 | 1.44 | 0.96-2.18 | 1.06 | 1.01-1.12 | 0% |  |  |
| Total sitting | 7 | 2.27 | 0.97-2.38 | 1.09 | 0.82-1.46 | 36% |  |  |
| TV-viewing time | 3 | 1.50 | 0.60-4.00 | 1.4 | 0.85-2.31 | 34% | 1.19 | **.55** |
| **Abbreviations:** RR relative risk, CI confidence interval, REML random effect model, BMI Body-Mass-Index, NA not applicable | | | | | | |  |  |
| *statistical difference in the subgroups |  |  |  |  |  |  |  |  |

| Table S13: Subgroup analysis of sedentary behavior and prostate cancer incidence | | | | | | |  |  |
| --- | --- | --- | --- | --- | --- | --- | --- | --- |
|  |  |  | **Largest study** | | **REML** | |  |  |
| **Variable** | **Number of included RRs** | **RR** | **95% CI** | **RR (high vs low SB)** | **95 % CI** | **I² (%)** | **Q (diff)** | **p (diff)*** |
| **Region** |  |  |  |  |  |  |  |  |
| North America | 2 | 0.98 | 0.91-1.05 | 0.97 | 0.93-1.02 | 0% |  |  |
| Europe | 10 | 1.67 | 1.02-2.73 | 1.17 | 1.01-1.12 | 38% |  |  |
| Asia | 2 | 1.23 | 0.96-1.58 | 1.12 | 0.97-1.41 | 0% | 9.30 | **0.01** |
| **Study design** |  |  |  |  |  |  |  |  |
| Case-control | 1 | 1.10 | 0.10-11.76 | 1.10 | 0.10-11.76 | NA |  |  |
| Cohort | 13 | 1.67 | 1.02-2.73 | 1.08 | 1.01-1.17 | 59% | 0.0001 | **.97** |
| **Adjusted for BMI** |  |  |  |  |  |  |  |  |
| No | 5 | 1.67 | 1.02-2.73 | 1.14 | 0.98-1.32 | 0% |  |  |
| Yes | 9 | 1.30 | 0.92-1.84 | 1.06 | 0.97-1.16 | 65% | 0.63 | **.42** |
| **Adjusted for smoking** |  |  |  |  |  |  |  |  |
| No | 5 | 1.30 | 0.92-1.84 | 1.07 | 0.93-1.22 | 30% |  |  |
| Yes | 9 | 1.67 | 1.02-2.73 | 1.09 | 0.99-1.19 | 65% | 0.05 | **.83** |
| **Adjusted for alcohol consumption** |  |  |  |  |  |  |  |  |
| No | 6 | 1.67 | 1.02-2.73 | 1.16 | 1.01-1.33 | 0% |  |  |
| Yes | 8 | 1.27 | 1.11-1.46 | 1.05 | 0.96-1.15 | 67% | 1.36 | **.24** |
| **Adjusted for family history of cancer** |  |  |  |  |  |  |  |  |
| No | 8 | 1.67 | 1.02-2.73 | 1.14 | 1.05-1.25 | 0% |  |  |
| Yes | 6 | 1.27 | 1.11-1.46 | 1.03 | 0.93-1.14 | 66% | 2.50 | **.11** |
| **Sedentary behavior** |  |  |  |  |  |  |  |  |
| Occupational sitting | 10 | 1.67 | 1.02-2.73 | 1.11 | 1.01-1.22 | 34% |  |  |
| Total sitting | 3 | 1.22 | 1.05-1.42 | 1.06 | 0.92-1.26 | 75% |  |  |
| TV-viewing time | 1 | 0.98 | 0.91-1.05 | 0.98 | 0.91-1.05 | NA | 4.60 | **.1** |
| **Abbreviations:** RR relative risk, CI confidence interval, REML random effect model, BMI Body-Mass-Index, NA not applicable | | | | | | |  |  |
| *statistical difference in the subgroups |  |  |  |  |  |  |  |  |

**Reference list of included and excluded systematic reviews**

1. Schmid D, Leitzmann MF. Television viewing and time spent sedentary in relation to cancer risk: a meta-analysis. *J Natl Cancer Inst*. 2014;106(7).

2. Ekelund U, Brown WJ, Steene-Johannessen J, et al. Do the associations of sedentary behaviour with cardiovascular disease mortality and cancer mortality differ by physical activity level? A systematic review and harmonised meta-analysis of data from 850 060 participants. *Br J Sports Med*. 2019;53(14):886-94.

3. Zhao R, Bu W, Chen Y, et al. The Dose-Response Associations of Sedentary Time with Chronic Diseases and the Risk for All-Cause Mortality Affected by Different Health Status: A Systematic Review and Meta-Analysis. *J Nutr Health Aging*. 2020;24(1):63-70.

4. Patterson R, McNamara E, Tainio M, et al. Sedentary behaviour and risk of all-cause, cardiovascular and cancer mortality, and incident type 2 diabetes: a systematic review and dose response meta-analysis. *Eur J Epidemiol*. 2018;33(9):811-29.

5. Biswas A, Oh PI, Faulkner GE, et al. Sedentary time and its association with risk for disease incidence, mortality, and hospitalization in adults: a systematic review and meta-analysis. *Ann Intern Med*. 2015;162(2):123-32.

6. Shen D, Mao W, Liu T, et al. Sedentary behavior and incident cancer: a meta-analysis of prospective studies. *PLoS One*. 2014;9(8):e105709.

7. Lynch BM, Shahid Mahmood, Terry Boyle. Sedentary Behaviour and Cancer. In: Leitzmann MF, Carmen Jochem, Daniela Schmid, editor. Sedentary behaviour epidemiology: Springer; 2018. p. 245-98.

8. Jochem C, Wallmann-Sperlich B, Leitzmann MF. The Influence of Sedentary Behavior on Cancer Risk: Epidemiologic Evidence and Potential Molecular Mechanisms. *Curr Nutr Rep*. 2019;8(3):167-74.

9. de Rezende LF, Rey-Lopez JP, Matsudo VK, et al. Sedentary behavior and health outcomes among older adults: a systematic review. *BMC Public Health*. 2014;14:333.

10. Thorp AA, Owen N, Neuhaus M, et al. Sedentary behaviors and subsequent health outcomes in adults a systematic review of longitudinal studies, 1996-2011. *Am J Prev Med*. 2011;41(2):207-15.

11. Proper KI, Singh AS, van Mechelen W, et al. Sedentary behaviors and health outcomes among adults: a systematic review of prospective studies. *Am J Prev Med*. 2011;40(2):174-82.

12. Xu C, Furuya-Kanamori L, Liu Y, et al. Sedentary Behavior, Physical Activity, and All-Cause Mortality: Dose-Response and Intensity Weighted Time-Use Meta-analysis. *J Am Med Dir Assoc*. 2019;20(10):1206-12.e3.

13. van Uffelen JG, Wong J, Chau JY, et al. Occupational sitting and health risks: a systematic review. *Am J Prev Med*. 2010;39(4):379-88.

14. Lynch BM. Sedentary behavior and cancer: a systematic review of the literature and proposed biological mechanisms. *Cancer Epidemiol Biomarkers Prev*. 2010;19(11):2691-709.

15. Lacombe J, Armstrong MEG, Wright FL, et al. The impact of physical activity and an additional behavioural risk factor on cardiovascular disease, cancer and all-cause mortality: a systematic review. *BMC Public Health*. 2019;19(1):900.

16. Katzmarzyk PT, Church TS, Craig CL, et al. Sitting time and mortality from all causes, cardiovascular disease, and cancer. *Med Sci Sports Exerc*. 2009;41(5):998-1005.

17. Moore SC, Gierach GL, Schatzkin A, et al. Physical activity, sedentary behaviours, and the prevention of endometrial cancer. *Br J Cancer*. 2010;103(7):933-8.

18. Chong F, Wang Y, Song M, et al. Sedentary behavior and risk of breast cancer: a dose-response meta-analysis from prospective studies. *Breast Cancer*. 2020.

19. Chan DSM, Abar L, Cariolou M, et al. World Cancer Research Fund International: Continuous Update Project-systematic literature review and meta-analysis of observational cohort studies on physical activity, sedentary behavior, adiposity, and weight change and breast cancer risk. *Cancer Causes Control*. 2019;30(11):1183-200.

20. Zhou Y, Zhao H, Peng C. Association of sedentary behavior with the risk of breast cancer in women: update meta-analysis of observational studies. *Ann Epidemiol*. 2015;25(9):687-97.

21. Lee J, Lee J, Lee DW, et al. Sedentary work and breast cancer risk: A systematic review and meta-analysis. *J Occup Health*. 2021;63(1):e12239.

22. Lee J. Physical activity, sitting time, and the risk of ovarian cancer: A brief research report employing a meta-analysis of existing. *Health Care Women Int*. 2019;40(4):433-58.

23. Biller VS, Leitzmann MF, Sedlmeier AM, et al. Sedentary behaviour in relation to ovarian cancer risk: a systematic review and meta-analysis. *Eur J Epidemiol*. 2021;36(8):769-80.

24. Mahmood S, MacInnis RJ, English DR, et al. Domain-specific physical activity and sedentary behaviour in relation to colon and rectal cancer risk: a systematic review and meta-analysis. *Int J Epidemiol*. 2017;46(6):1797-813.

25. Ma P, Yao Y, Sun W, et al. Daily sedentary time and its association with risk for colorectal cancer in adults: A dose-response meta-analysis of prospective cohort studies. *Medicine (Baltimore)*. 2017;96(22):e7049.

26. Cong YJ, Gan Y, Sun HL, et al. Association of sedentary behaviour with colon and rectal cancer: a meta-analysis of observational studies. *Br J Cancer*. 2014;110(3):817-26.

27. Wang J, Huang L, Gao Y, et al. Physically active individuals have a 23% lower risk of any colorectal neoplasia and a 27% lower risk of advanced colorectal neoplasia than their non-active counterparts: systematic review and meta-analysis of observational studies. *Br J Sports Med*. 2019.

28. Berger FF, Leitzmann MF, Hillreiner A, et al. Sedentary Behavior and Prostate Cancer: A Systematic Review and Meta-Analysis of Prospective Cohort Studies. *Cancer Prev Res (Phila)*. 2019;12(10):675-88.

29. Swain CTV, Nguyen NH, Eagles T, et al. Postdiagnosis sedentary behavior and health outcomes in cancer survivors: A systematic review and meta-analysis. *Cancer*. 2020;126(4):861-9.
